# Performance of a Sepsis Prediction Model Across Different Sepsis Definitions

**DOI:** 10.1001/jamanetworkopen.2026.5599

**Published:** 2026-04-07

**Authors:** Sayon Dutta, Reid McMurry, Michael C. Tasi, Lisette Dunham, Dustin S. McEvoy, Timothy Stump, Michael Filbin, Chanu Rhee

**Affiliations:** 1 Department of Emergency Medicine, Massachusetts General Hospital, Boston; 2 Mass General Brigham Digital Health, Boston, Massachusetts; 3 Harvard Medical School, Boston, Massachusetts; 4 Department of Emergency Medicine, Boston Medical Center, Boston, Massachusetts; 5 Trinity Health, Grand Rapids, Michigan; 6 Division of Infectious Diseases, Department of Medicine, Brigham and Women’s Hospital, Boston, Massachusetts; 7 Department of Population Medicine, Harvard Medical School/Harvard Pilgrim Health Care Institute, Boston, Massachusetts

## Abstract

**Importance:**

Early detection of sepsis improves clinical outcomes, but the Early Detection of Sepsis Model, version 1 (Epic Systems Corp) has shown poor performance. Comparing sepsis models is complicated by varying outcome definitions and limited generalizability outside the development site.

**Objective:**

To evaluate the Early Detection of Sepsis Model, version 2 using multiple standard sepsis definitions.

**Design, Setting, and Participants:**

This diagnostic study included all adult (aged ≥18 years) encounters from emergency departments, inpatient units, intensive care units, and perioperative areas across 9 acute care hospitals between March 17 and August 31, 2024. The locally trained gradient-boosted tree ensemble sepsis model, incorporating patient demographics, vital signs, laboratory results, medication administration, and other clinical features, generated predictions every 15 minutes. Model performance was evaluated against 3 electronically computable sepsis definitions: the Third International Consensus Definitions for Sepsis and Septic Shock (Sepsis-3); Centers for Medicare & Medicaid Services Severe Sepsis and Septic Shock: Management Bundle (SEP-1); and the Centers for Disease Control and Prevention Adult Sepsis Event (ASE).

**Exposure:**

Silent deployment of the sepsis model.

**Main Outcomes and Measures:**

Model performance metrics included discrimination, as measured using the area under the receiver operating characteristic curve (AUROC), and precision, as measured by the area under the precision-recall curve (AUPRC). Test characteristics and potential lead-time warning were evaluated over a range of model score thresholds.

**Results:**

Among 198 494 patient encounters (median [IQR] age, 55 [36-71] years; 54.8% female) the incidence of sepsis varied by outcome definition. A total of 5832 encounters (2.9%) met the Sepsis-3 definition, 2366 (1.2%) met the SEP-1 definition, and 3881 (2.0%) met the ASE definition. The AUROC and AUPRC were, respectively, 0.89 (95% CI, 0.89-0.90) and 0.24 (95% CI, 0.23-0.25) for Sepsis-3, 0.94 (95% CI, 0.94-0.94) and 0.16 (95% CI, 0.15-0.17) for SEP-1, and 0.85 (95% CI, 0.85-0.86) and 0.11 (95% CI, 0.11-0.12) for ASE. In the overall study population, the Youden top left threshold for the Sepsis-3 outcome definition was 9, which yielded a positive predictive (PPV) of 11.4% (95% CI, 11.1%-11.7%) and median lead time of 3.4 hours (IQR, 0.9-22.3 hours). The SEP-1 had a PPV of 6.8 (95% CI, 6.6-7.1 9.9) and median (IQR) lead time of 4.5 (4.3-23.1) hours, and the ASE had a PPV of 5.9 (95% CI, 5.7-6.0) and median (IQR) lead time of 1.4 (0.5-14.8) hours.

**Conclusions and Relevance:**

This cohort study found that the locally trained sepsis model showed moderate predictive performance, with discrimination, precision, and lead time varying considerably by the sepsis definition applied. Although the model provided some advance warning before sepsis onset, a high false-positive rate may limit its clinical utility without careful threshold selection and tailored implementation.

## Key Points

**Question:**

How does an electronic health record locally trained early detection of sepsis model perform across commonly used, electronically computable sepsis definitions?

**Findings:**

In this diagnostic study of 198 494 patient encounters, model performance varied substantially by sepsis definition (Third International Consensus Definitions for Sepsis and Septic Shock [Sepsis-3], Severe Sepsis and Septic Shock: Management Bundle, and Adult Sepsis Event) with overall good discrimination but modest and variable precision, positive predictive value, and lead time. Performance was highest for Sepsis-3.

**Meaning:**

These findings suggest that the locally trained early detection of sepsis model may provide moderate predictive accuracy and early warning for sepsis, but high false-positive rates and variability across definitions underscore the need for careful calibration and tailored implementation.

## Teaser

This diagnostic study evaluates an early detection of sepsis prediction model using multiple standard sepsis definitions among adults in the northeastern US.

## Introduction

Sepsis is a leading cause of morbidity and mortality worldwide.^1,2^ In the US, there are an estimated 1.7 million adult hospitalizations annually due to sepsis, with Medicare patients alone responsible for costs greater than $40 billion.^1,3^ Early recognition and initiation of treatment of sepsis is key to survival, with each 1-hour delay in treatment associated with a 4% increase in mortality.^4,5,6,7,8,9^ Machine learning models that can predict sepsis have the potential to allow for earlier treatment initiation. One of the most widely deployed models has been the Early Detection of Sepsis Model (Epic System Corp). Version 1 of this electronic health record (EHR)–based sepsis model is a penalized regression model with static coefficients. However, independent evaluations have found this model to have poor performance, raising concerns about its utility in clinical settings.^10,11,12,13,14,15^

Version 2 of the model was subsequently released in November 2022, but evaluations of this version remain limited to date.^16^ Version 2 of the model is a locally trained gradient-boosted tree ensemble trained on the Third International Consensus Definitions for Sepsis and Septic Shock (Sepsis-3) outcome definition.^17^ The model uses features that include patient age, diagnoses, laboratory values, vital signs, nursing flowsheet assessments, medication administration, procedures, and prior use. Full details of the model’s development strategy are available to the vendor’s customers.^18^ The sepsis model is especially worthy of performance evaluation, as it is perhaps the most widely implemented machine learning model in health care. The vendor reported 125 health care organizations that are live on version 1 and 78 on version 2 as of May 2025.

However, evaluation of sepsis detection machine learning models is complicated by the use of heterogeneous sepsis outcome definitions across studies, making direct comparisons of model performance difficult.^10,11,12,13,14,15,16,19,20^ Standardized, multidefinition evaluations are needed to better characterize model performance across contexts.

We performed a silent prospective performance evaluation of a locally trained instantiation of the sepsis prediction model before consideration of clinical implementation. The outcome was the comparison of the model’s performance on 3 established sepsis definitions: Sepsis-3; the Centers for Medicare & Medicaid Services Severe Sepsis and Septic Shock: Management Bundle (SEP-1); and the Centers for Disease Control and Prevention Adult Sepsis Event (ASE).^17,21,22^ These definitions reflect the range of clinical applications: Sepsis-3 is most commonly used in clinical research and model development, SEP-1 serves as the basis for hospital quality reporting in the US, and ASE was designed for epidemiologic surveillance and public health reporting and is now being used as the foundation for a proposed national hospital mortality outcome measure.^23,24^

## Methods

### Patients, Design, and Setting

This diagnostic study included patients aged 18 years or older who presented to an acute care hospital within the Mass General Brigham (MGB) health care system between March 17 and August 31, 2024. The MGB Institutional Review Board approved this study with a waiver of informed consent as it posed minimal risk to human participants. The study followed the Transparent Reporting of a Multivariable Prediction Model for Individual Prognosis or Diagnosis Plus Artificial Intelligence (TRIPOD+AI) reporting guideline.^25^

The MGB health care system encompasses 2 quaternary-care academic centers, 5 community hospitals, and 2 critical access hospitals. Eligible hospital encounters included those that began in the emergency department (ED) or were direct admissions, excluding those that began in endoscopy; were direct admissions to a psychiatric service; or were ED visits with direct transfers to labor and delivery. Emergency department encounters that were discharged without admission were included. Encounters with hospital stays of less than 1 hour were excluded. All study sites use the same EHR.

### EHR Predictive Model Localization and Implementation

Version 2 of the sepsis model was initially developed using data from 3 non-MGB health systems. For local implementation, the vendor defined the model architecture, inputs, and hyperparameters. We mapped input features to local equivalents and locally trained the model on the Sepsis-3 outcome using approximately 250 000 encounters for adult patients seen between January 1 and May 31, 2023, in our health care system. There was no overlap between the training population and the cohort used in this study. The model handled missing values natively. Null values for oxygen delivery were replaced by room air, and all other missing features were processed without imputation. The model prospectively generated predictions (0-100) every 15 minutes for all adult patients in the hospital, which were stored in the EHR but not shown to clinicians or used in clinical decision support. Model scores did not directly correlate with the positive predictive value (PPV), ie, a score of 60 did not equate to a 60% risk of sepsis. The model version, training parameters, and calibration were unchanged during the study period.

### Measurements

All data were extracted from the EHR data warehouse using structured query language. The queries captured relevant data on patient demographics, unit type within the hospital, laboratory values, vital signs, and predictive model scores. Patient race (Asian, Black or African American, White, multiracial or other [American Indian or Alaska Native or Native Hawaiian or Pacific Islander], or unknown) and ethnicity (Hispanic, non-Hispanic, or unknown) were self-reported and extracted from the EHR and collected to evaluate model performance within the strata race and ethnicity. Missing demographic variables were reported as unavailable. Encounter type defined the type of hospitalization; however, some encounters defined as ED only without hospitalization may have been transferred back to an inpatient rehabilitation facility or a different ED. Accuracy of the structured query language queries and sepsis outcome definitions was confirmed through random review of 35 patient records.

### Outcome Definition

The primary outcome was sepsis model performance under the Sepsis-3 definition, the SEP-1–based computable definition, and the ASE definition.^17,21,22,23,24^ Time zero was defined as the earliest qualifying time point per definition, as follows: culture or antibiotics for Sepsis-3; 2 or more systemic inflammatory response syndrome criteria with organ dysfunction for SEP-1; and culture, antibiotics, and organ dysfunction for ASE. Full definitions for each outcome and their corresponding time zero are listed in eTable 1 in Supplement 1, as well as how the computable SEP-1 definition differs from the SEP-1 quality measure.

### Statistical Analysis

The minimum sample size required for this study was calculated using the method described by Riley et al^26^ (details provided in the eMethods in Supplement 1). The study population is described using counts and proportions for categorical variables and medians and IQRs for continuous variables. The time between hospital arrival and the first sepsis time zero in the encounter is reported, stratified by encounter type.

The model performance was evaluated at the encounter and prediction level (within specific horizons of 8 hours and 24 hours) using the area under the receiver operating characteristic curve (AUROC), area under the precision-recall curve (AUPRC), estimated calibration error (ECE), decision curve analysis, and lead-time analysis. Confidence intervals were bootstrapped by resampling at the encounter level and accounted for clustering at the patient level. Precision and recall over a range of model thresholds per outcome definition are reported. Model performance at predefined thresholds favoring high recall (≥0.8), high precision (≥0.15), and Youden top left (the point on the receiver operating characteristic curve that maximizes the trade-off between sensitivity and specificity) is reported, including the false-positive rate and number of encounters requiring evaluation to detect 1 true-positive encounter. Details of this evaluation framework for classification models, previously used in other studies, are available in the eMethods in Supplement 1.^12,27,28,29,30^ Prediction-level model performance was evaluated by whether the patient was in the ED, on the ward, or in the intensive care unit (ICU) when the prediction was created.

Model performance was calculated within strata of population subgroups based on demographics, encounter type, and hospital type. Performance drift was evaluated by calculating the AUROC and AUPRC per week during the study period. In recognition of the limited clinical utility offered by a model that recognizes sepsis after treatment has already been initiated, a sensitivity analysis was performed using the framework described by Kamran et al^10^ to evaluate the performance of the model prior to initiation of treatment. For this analysis, we filtered predictions to retain only those that occurred prior to sepsis-related clinical actions, specifically the ordering of lactic acid, blood cultures, or intravenous antibiotics. The analyses were performed using R, version 4.4.1 (R Foundation for Statistical Computing) and Python, version 3.12 (Python Software Foundation).^31^

## Results

### Patient Characteristics

Over the 6-month study period, 207 489 hospital encounters met the inclusion criteria. We excluded 8995 encounters due to a hospital length of stay less than 1 hour. The final analysis included 198 494 hospital encounters (median [IQR] patient age, 55 [36-71 years]; 54.8% female and 45.2% male; 3.5% identifying as Asian, 11.9% as Black or African American, 69.6% as White, 1.1% as multiracial or other, and 13.9% as unknown race; 16.0% identifying as Hispanic, 80.4% as non-Hispanic, and 3.5% as unknown ethnicity) (eTable 2 in Supplement 1). A total of 68.1% patients were seen in the ED and discharged without admission to the same hospital, 21.8% were admitted from the ED to the hospital, and 4.9% were admitted to the hospital after an elective operative procedure.

The sepsis outcome incidence in the overall study population was 2.9% (5832 encounters) using the Sepsis-3 definition, 1.2% (2366 encounters) using the SEP-1 definition, and 2.0% (3881 encounters) using the ASE definition (Table). Patients with sepsis compared with those without sepsis were older (median [IQR] age, 69 [57-79] vs 55 [35-71] years for Sepsis-3, 69 [58-79] vs 55 [36-71] years for SEP-1, and 68 [58-78] vs 55 [35-71] years for ASE) and admitted to the hospital or seen at the academic hospitals (57.5% vs 42.6% for Sepsis-3, 49.7% vs 43.0% for SEP-1, and 62.5% vs 42.7% for ASE) (eTable 3 in Supplement 1). Most patients with sepsis arrived via the ED (Sepsis-3, 64.2%; SEP-1, 82.4%; ASE, 77.8%) rather than direct admission (Sepsis-3, 15.8%; SEP-1, 13.4%; ASE, 16.9%) or admission following an operative procedure (Sepsis-3, 3.6%; SEP-1, 2.3%; ASE, 5.1%). Incidence of in-hospital mortality also varied by outcome definition (Sepsis-3, 519 of 5832 [14.2%]; SEP-1, 519 of 2366 [21.9%]; ASE, 727 of 3881 [18.7%]).

**Table.** Early Detection of Sepsis Predictive Model Encounter-Level Performance

Population	ASE outcome definition			SEP-1 outcome definition			Sepsis-3 outcome definition		
	No. detected/total No. (%)	AUROC	AUPRC	No. detected/total No. (%)	AUROC	AUPRC	No. detected/total No. (%)	AUROC	AUPRC
Overall	3881/198 494 (2.0)	0.85 (0.84-0.86)	0.11 (0.11-0.12)	2366/198 494 (1.2)	0.94 (0.94-0.94)	0.16 (0.15-0.17)	5832/198 494 (2.9)	0.89 (0.89-0.90)	0.24 (0.23-0.25)
Sex									
Female	1677/108 763 (1.5)	0.86 (0.85-0.87)	0.11 (0.10-0.12)	1075/108 763 (1.0)	0.94 (0.93-0.95)	0.15 (0.14-0.17)	2605/108 763 (2.4)	0.90 (0.90-0.91)	0.23 (0.22-0.25)
Male	2204/89 731 (2.5)	0.84 (0.83-0.85)	0.12 (0.11-0.13)	1291/89 731 (1.4)	0.94 (0.93-0.94)	0.16 (0.15-0.18)	3227/89 731 (3.6)	0.88 (0.88-0.89)	0.24 (0.23-0.26)
Race									
Asian	146/6,940 (2.1)	0.87 (0.84-0.90)	0.14 (0.11-0.19)	89/6940 (1.3)	0.96 (0.95-0.97)	0.22 (0.15-0.31)	209/6940 (3.0)	0.91 (0.89-0.92)	0.25 (0.20-0.30)
Black or African American	336/23 709 (1.4)	0.89 (0.87-0.90)	0.11 (0.09-0.14)	192/23 709 (0.8)	0.96 (0.95-0.96)	0.15 (0.12-0.19)	495/23 709 (2.1)	0.92 (0.90-0.93)	0.24 (0.21-0.28)
White	2992/138 082 (2.2)	0.84 (0.83-0.84)	0.11 (0.10-0.12)	1842/138 082 (1.3)	0.93 (0.93-0.94)	0.16 (0.15-0.17)	4520/138 082 (3.3)	0.88 (0.88-0.89)	0.23 (0.22-0.25)
Multiracial or other^a^	21/2268 (0.9)	0.89 (0.85-0.92)	0.05 (0.03-0.11)	10/2268 (0.4)	0.94 (0.91-0.96)	0.05 (0.02-0.17)	36/2268 (1.6)	0.90 (0.85-0.94)	0.16 (0.09-0.32)
Unknown	386/27 495 (1.4)	0.88 (0.87-0.89)	0.12 (0.10-0.16)	233/27 495 (0.8)	0.96 (0.95-0.96)	0.14 (0.12-0.18)	572/27 495 (2.1)	0.92 (0.91-0.93)	0.27 (0.24-0.31)
Ethnicity									
Hispanic	360/31 810 (1.1)	0.90 (0.88-0.91)	0.12 (0.10-0.16)	233/31 810 (0.7)	0.96 (0.95-0.97)	0.15 (0.12-0.20)	527/31 810 (1.7)	0.92 (0.91-0.93)	0.23 (0.20-0.26)
Not Hispanic	3299/159 669 (2.1)	0.84 (0.84-0.85)	0.11 (0.10-0.12)	2014/159 669 (1.3)	0.94 (0.93-0.94)	0.16 (0.15-0.17)	5004/159 669 (3.1)	0.89 (0.88-0.89)	0.23 (0.22-0.24)
Unknown	222/7015 (3.2)	0.83 (0.80-0.85)	0.13 (0.11-0.17)	119/7015 (1.7)	0.94 (0.92-0.95)	0.19 (0.15-0.26)	301/7015 (4.3)	0.91 (0.89-0.92)	0.34 (0.29-0.42)
ESI									
Immediate	146/1 245 (11.7)	0.65 (0.61-0.69)	0.17 (0.14-0.22)	78/1245 (6.3)	0.73 (0.68-0.78)	0.13 (0.10-0.19)	227/1245 (18.2)	0.74 (0.71-0.77)	0.34 (0.30-0.41)
Emergent	1752/53 647 (3.3)	0.82 (0.82-0.83)	0.13 (0.13-0.15)	1250/53 647 (2.3)	0.91 (0.91-0.92)	0.20 (0.19-0.22)	2674/53 647 (5.0)	0.86 (0.86-0.87)	0.26 (0.24-0.28)
Urgent	1076/96 182 (1.1)	0.85 (0.84-0.86)	0.07 (0.07-0.09)	642/96 182 (0.7)	0.95 (0.94-0.96)	0.15 (0.13-0.17)	1738/96 182 (1.8)	0.88 (0.87-0.88)	0.15 (0.14-0.17)
Less urgent	30/25 534 (0.1)	0.87 (0.80-0.92)	0.03 (0.01-0.12)	15/25 534 (0.1)	0.99 (0.98-1.00)	0.14 (0.05-0.30)	30/25 534 (0.1)	0.95 (0.93-0.98)	0.04 (0.02-0.10)
Encounter type									
Direct hospital admission	655/8983 (7.3)	0.67 (0.64-0.69)	0.13 (0.12-0.15)	317/8983 (3.5)	0.82 (0.80-0.85)	0.13 (0.11-0.16)	919/8983 (10.2)	0.80 (0.79-0.82)	0.33 (0.30-0.36)
ED only, without admission	6/135 163 (<0.1)	0.98 (0.97-1.00)	0.02 (0.00-0.13)	44/135 163 (<.1)	0.96 (0.93-0.98)	0.04 (0.02-0.09)	374/135 163 (0.3)	0.92 (0.91-0.94)	0.07 (0.06-0.10)
ED to hospitalization	3020/43 247 (7.0)	0.69 (0.68-0.70)	0.15 (0.14-0.16)	1950/43 247 (4.5)	0.84 (0.84-0.85)	0.20 (0.19-0.21)	4328/43 247 (10.0)	0.75 (0.74-0.76)	0.26 (0.25-0.28)
OB to hospitalization	1/1397 (0.1)	0.04 (0.03-0.05)	0.00 (0.00-0.00)	0/1397 (0)	NA	NA	1/1397 (0.1)	0.80 (0.78-0.81)	0.00 (0.00-0.01)
OR to hospitalization	199/9704 (2.1)	0.60 (0.55-0.64)	0.06 (0.04-0.09)	55/9704 (0.6)	0.91 (0.88-0.94)	0.07 (0.04-0.11)	210/9704 (2.2)	0.84 (0.80-0.86)	0.19 (0.14-0.24)
Hospital type									
Academic medical center	2427/85 513 (2.8)	0.82 (0.81-0.83)	0.12 (0.11-0.13)	1177/85 513 (1.4)	0.93 (0.92-0.93)	0.14 (0.13-0.15)	3354/85 513 (3.9)	0.88 (0.87-0.88)	0.25 (0.24-0.26)
Community hospital	1423/101 766 (1.4)	0.87 (0.86-0.88)	0.10 (0.09-0.11)	1170/101 766 (1.1)	0.94 (0.94-0.95)	0.20 (0.18-0.22)	2376/101 766 (2.3)	0.90 (0.90-0.91)	0.23 (0.22-0.25)
Critical access hospital	31/11 215 (0.3)	0.89 (0.82-0.94)	0.03 (0.02-0.06)	19/11 215 (0.2)	0.97 (0.95-0.99)	0.10 (0.04-0.24)	102/11 215 (0.9)	0.89 (0.86-0.92)	0.12 (0.09-0.18)

Abbreviations: ASE, Centers for Disease Control and Prevention Adult Sepsis Event; AUPRC, area under the precision-recall curve; AUROC, area under the receiver operating characteristic curve; ED, emergency department; ESI, emergency severity index; OB, obstetrics; OR, operating room; SEP-1, Centers for Medicare & Medicaid Services Severe Sepsis and Septic Shock: Management Bundle; Sepsis-3, Third International Consensus Definitions for Sepsis and Septic Shock.

^a^ Included American Indian or Alaska Native or Native Hawaiian or Pacific Islander.

The time between hospital arrival and sepsis time zero varied by sepsis outcome definition (median [IQR], 4.1 [1.2-25.2] hours for Sepsis-3, 5.8 [1.8-31.3] hours for SEP-1, and 1.7 [0.7-11.6] hours for ASE) (eFigure 1 in Supplement 1). The arrival to time zero varied by encounter type, for each outcome definition. For example, for Sepsis-3, the time between arrival to time zero was longer for direct admissions (median [IQR], 35.0 [3.9-170.9] hours) compared with admissions from the ED (median [IQR], 2.9 [1.0-10.3] hours).

### Model Performance

#### Encounter-Level Performance

**Figure 1.**
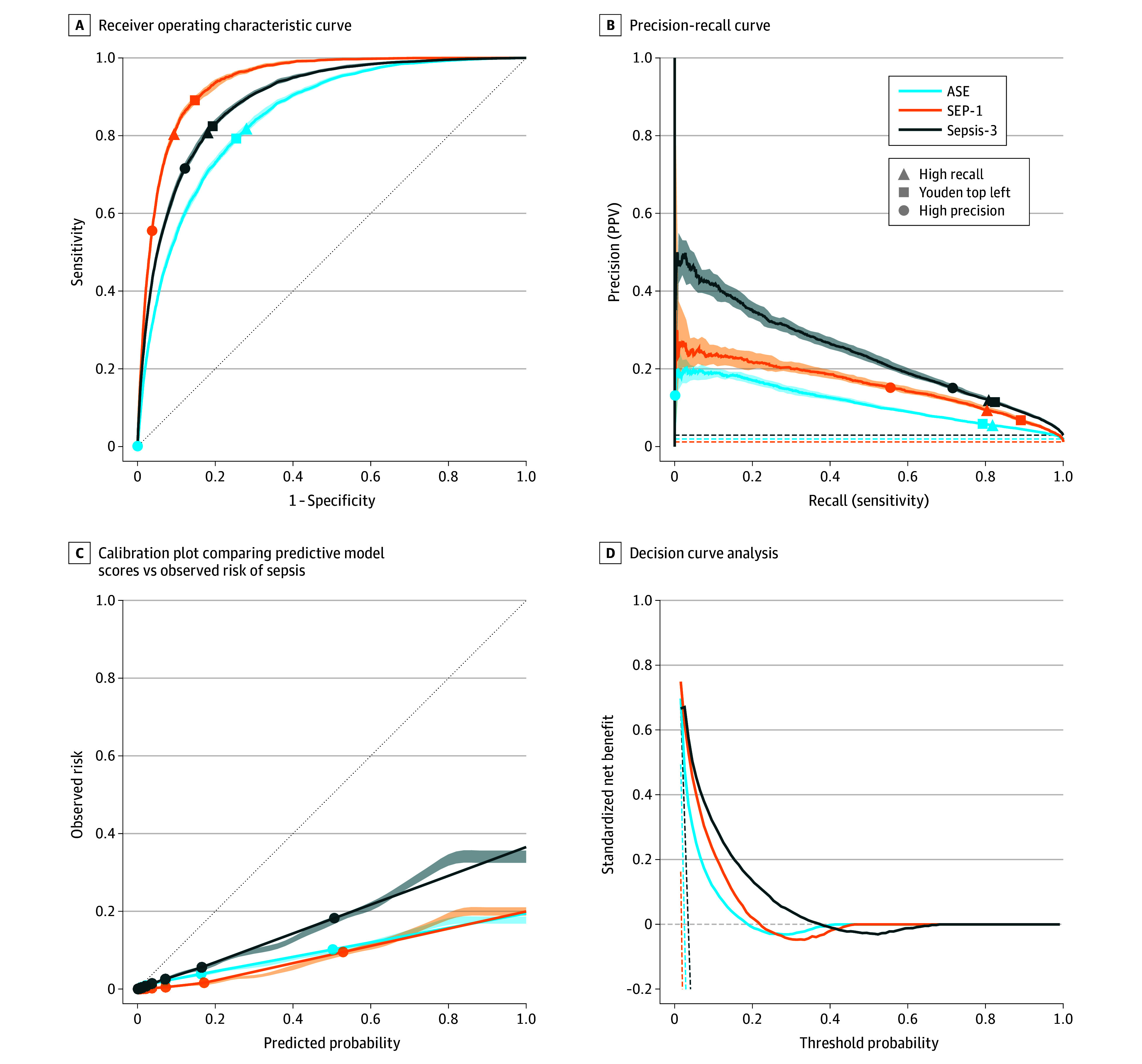
Line Graphs of Encounter-Level Model Performance of Different Sepsis Outcome Definitions A, The diagonal dashed line represents a random model. B, The dashed lines represent the incidence per sepsis outcome. C, The diagonal dashed line represents perfect calibration, and the circles show the average observed and predicted risk for each decile of the predictive score distribution (normalized to 0-1). D, Decision curves above the treat all lines (vertical dashed lines) and treat none line (horizontal dashed line) represent positive standardized net benefit. Shading indicates the 95% CI. Specific thresholds, precision, recall, false-positive rates, number of encounters needed to detect 1 true-positive encounter, lead times, and total number of encounters are presented in eTable 4 in Supplement 1 for each sepsis outcome definition. ASE indicates Centers for Disease Control and Prevention Adult Sepsis Event; SEP-1, Centers for Medicare & Medicaid Services Severe Sepsis and Septic Shock: Management Bundle; Sepsis-3, Third International Consensus Definitions for Sepsis and Septic Shock.

Among the 3 sepsis outcomes, the model had the highest discrimination for SEP-1 (AUROC, 0.94; 95% CI, 0.94-0.94), followed by Sepsis-3 (AUROC, 0.89; 95% CI, 0.89-0.90) and ASE (AUROC, 0.85; 95% CI, 0.85-0.86) (Figure 1A). The Sepsis-3 outcome had the highest precision (AUPRC, 0.24; 95% CI, 0.23-0.25) compared with SEP-1 (AUPRC, 0.16; 95% CI, 0.15-0.17) and ASE (AUPRC, 0.11; 95% CI, 0.11-0.12) (Figure 1B). Variations in incidence per sepsis outcome definition affect the PPV and therefore AUPRC, but the AUROC is incidence independent.

The performance of the model varied among population subgroups based on the incidence of sepsis. As incidence of sepsis increased with triage acuity (emergency severity index), AUROC declined, and AUPRC remained stable or improved. The Sepsis-3 outcome had the lowest ECE of 0.05, suggesting better alignment between predicted and observed risk compared with SEP-1 (ECE, 0.07) and ASE (ECE, 0.06) (Figure 1C). Decision curve analysis showed that the model provided higher standardized net benefit across a range of threshold probabilities for the SEP-1 and Sepsis-3 outcome definitions compared with the ASE definition (Figure 1D). Temporal performance drift was not detected over the study period (eFigure 2 in Supplement 1).

#### Prediction-Level Performance

The prediction-level model performance was better for the SEP-1 and Sepsis-3 outcomes compared with ASE. The SEP-1 definition achieved the best prediction-level performance with a time horizon of 8 hours (AUROC, 0.86; 95% CI, 0.86-0.87) (eFigure 3A in Supplement 1). The Sepsis-3 outcome within 8 hours of prediction had an AUROC of 0.84 (95% CI, 0.84-0.85). Sepsis-3 showed the highest precision (AUPRC, 0.05; 95% CI, 0.05-0.05) when using a 24-hour time horizon from each prediction, while all models exhibited low absolute precision due to class imbalance secondary to low per-prediction horizon sepsis incidence (eFigure 3B in Supplement 1). A sensitivity analysis was used to evaluate the prediction-level performance based on the unit type at the time of prediction (eFigures 4-6 in Supplement 1). The Sepsis-3 outcome with a horizon of 8 hours had an AUROC of 0.90 (95% CI, 0.89-0.90) in the ED, 0.82 (95% CI, 0.82-0.83) on inpatient wards, and 0.76 (95% CI, 0.75-0.77) in the ICU.

#### Performance Prior to Treatment Initiation

**Figure 2.**
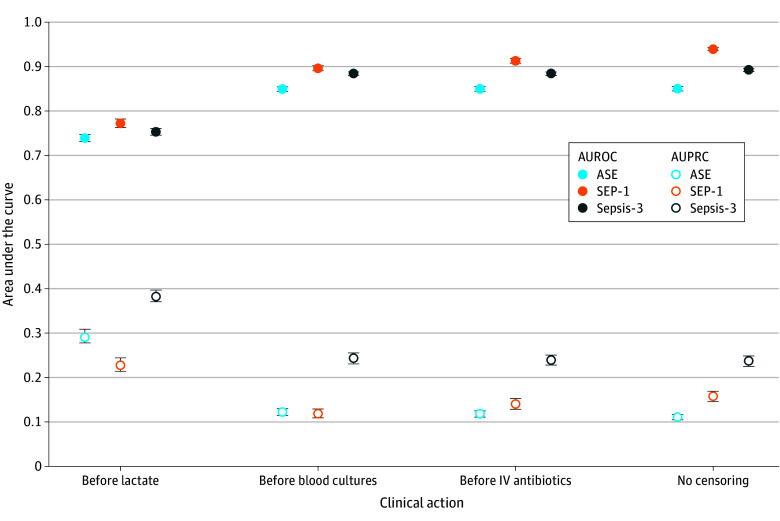
Dot-and-Whisker Plots of Model Performance With Respect to Sepsis Treatments The plots compare overall model performance with predictions made before sepsis clinical actions. Error bars indicate the 95% CI. ASE indicates Centers for Disease Control and Prevention Adult Sepsis Event; AUPRC indicates area under the precision-recall curve; AUROC, area under the receiver operating characteristic curve; IV, intravenous; SEP-1, Centers for Medicare & Medicaid Services Severe Sepsis and Septic Shock: Management Bundle; Sepsis-3, Third International Consensus Definitions for Sepsis and Septic Shock.

For the SEP-1 outcome, there was a small decrease in AUROC when censoring predictions to before orders for blood cultures and intravenous antibiotics; however, no decrease in the AUROC or AUPRC was seen for the Sepsis-3 or ASE outcomes (Figure 2). For the Sepsis-3 outcome, censoring predictions to before the first lactic acid order reduced the AUROC to 0.75 (95% CI, 0.75-0.76) while raising the AUPRC to 0.38 (95% CI, 0.37-0.40).

#### Classification Plot and Lead Time

**Figure 3.**
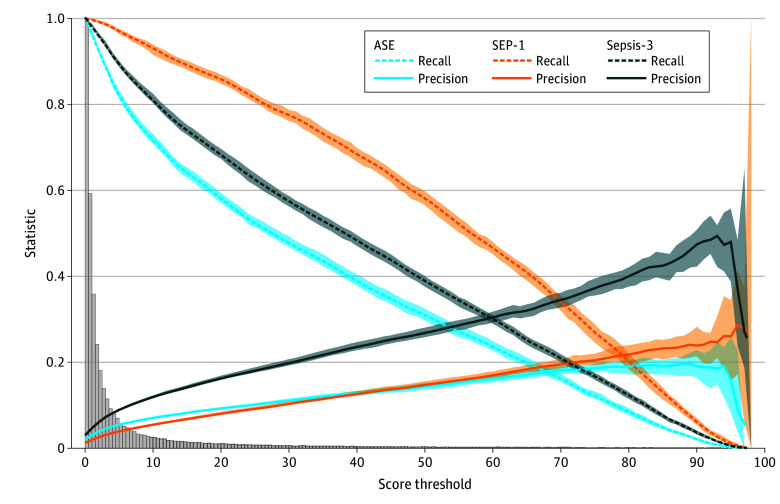
Line Plot and Histogram of Test Characteristics of the Sepsis Model by the Highest Predictive Model Score Within the Encounter Before the Sepsis Outcome Time Zero or Hospital Discharge Higher thresholds indicate greater precision but less recall. The histogram shows the maximum predictive model scores per patient encounter. The statistical values for each score threshold are provided in eTable 5 in Supplement 1. Shading indicates the 95% CI. ASE indicates Centers for Disease Control and Prevention Adult Sepsis Event; SEP-1, Centers for Medicare & Medicaid Services Severe Sepsis and Septic Shock: Management Bundle; Sepsis-3, Third International Consensus Definitions for Sepsis and Septic Shock.

**Figure 4.**
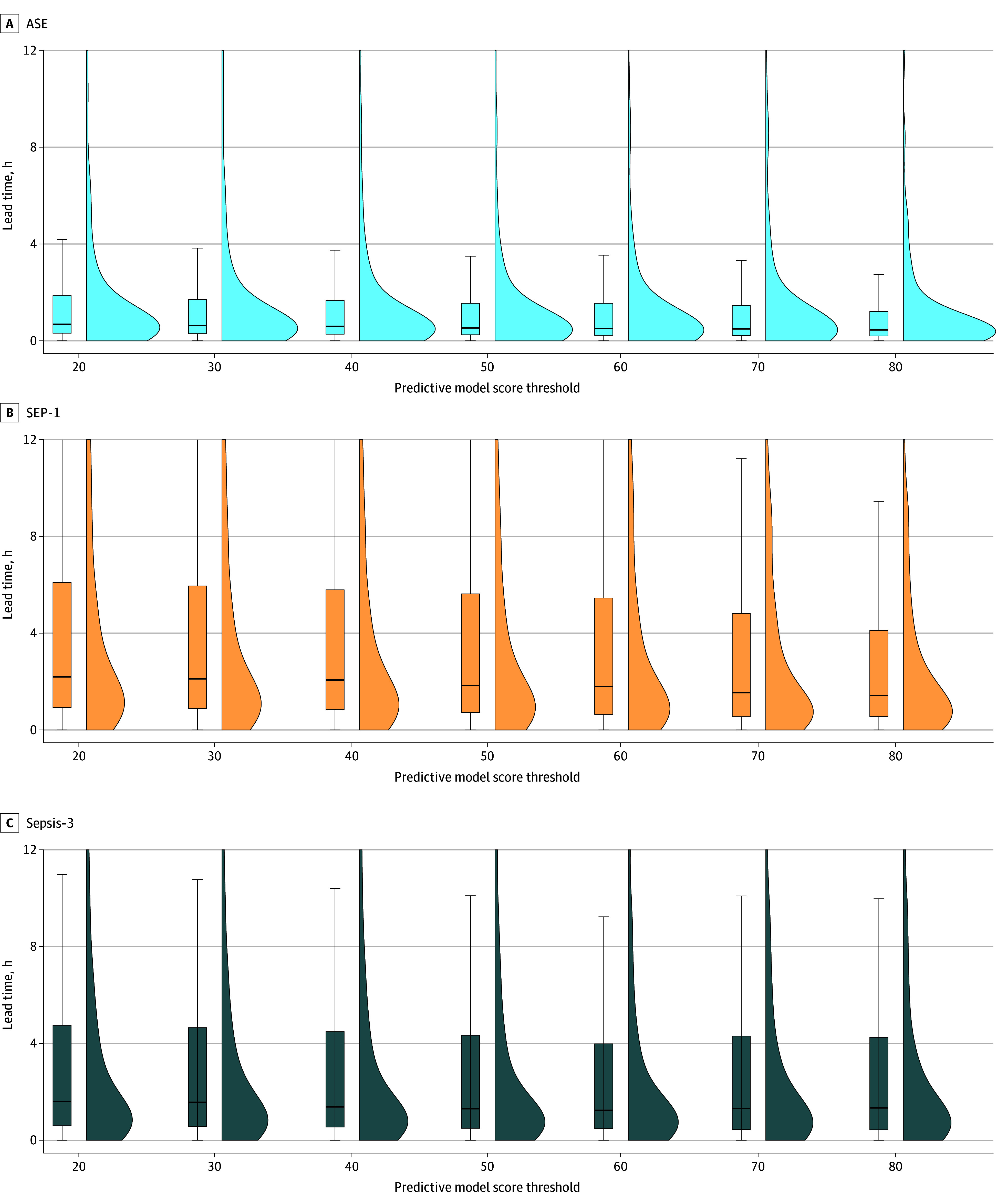
Box-and-Whisker and Density Plots of Lead Time Between the Sepsis Model Reaching a Score Threshold and Sepsis Time Zero The plots display how much warning before the sepsis outcome time zero is reached that the model would provide over a range of thresholds. The horizontal bar inside the boxes indicates the median, and the lower and upper ends of the boxes are the first and third quartiles. The whiskers indicate values within 1.5× the IQR from the upper or lower quartile. ASE indicates Centers for Disease Control and Prevention Adult Sepsis Event; SEP-1, Centers for Medicare & Medicaid Services Severe Sepsis and Septic Shock: Management Bundle; Sepsis-3, Third International Consensus Definitions for Sepsis and Septic Shock.

The sepsis model had higher precision on the Sepsis-3 outcome across all model score thresholds compared with the ASE and SEP-1 definitions in the overall population, as well as stratified by encounter type (Figure 3; eTable 4 and eFigure 7 in Supplement 1). In the overall study population, the Youden top left threshold for the Sepsis-3 outcome was 9, which yielded a precision of 11.4% (95% CI, 11.1%-11.7%), recall of 82.4% (95% CI, 81.8%-83.8%), false-positive rate of 19.3% (95% CI, 19.2%-19.6%), median lead time of 3.4 hours (IQR, 0.9-22.4 hours), and 8.7 (95% CI 8.5-9.0) encounters needing evaluation to identify 1 true-positive sepsis outcome (Figure 4; eTable 5 in Supplement 1). The SEP-1 had a PPV of 6.8 (95% CI, 6.6-7.1) and median (IQR) lead time of 4.5 (4.3-23.1) hours, and the ASE had a PPV of 5.9 (95% CI, 5.7-6.0) and median (IQR) lead time of 1.4 (0.5-14.8) hours (eTable 4 in Supplement 1).

At a threshold of 9, 21.2% of encounters met or exceeded the threshold. Stratified model performance showed different Youden top left thresholds based on encounter type (eFigures 8-10 in Supplement 1). For example, for admissions from the ED, the best-balanced threshold was 18, yielding a precision of 19.5% (95% CI, 18.8%-20.0%), recall of 69.4% (95% CI, 68.1%-70.8%), and 5.1 (95% CI, 5.0-5.3) encounters needing evaluation, although 35.7% (15 419 of 43 247) of encounters would reach this threshold.

## Discussion

This diagnostic study evaluating a locally trained instantiation of a vendor-specified sepsis model found that the performance of the model varied substantially depending on the sepsis outcome definition used. While the model achieved good discrimination for the Sepsis-3 outcome, the definition it was trained on (AUROC, 0.89; AUPRC, 0.24), its performance was less robust when evaluated against alternative definitions. Importantly, lead time prior to sepsis onset was modest (median of 3.4 hours for Sepsis-3), and the model showed a high false-positive rate. These findings underscore both the potential and limitations of machine learning models for early sepsis detection, particularly when applied across heterogeneous clinical definitions and care settings.

In subgroup analyses stratified by care location, model discrimination decreased with increasing patient acuity, with the highest AUROC observed in the ED and progressively lower performance on the inpatient wards and in the ICU. This pattern reflects risk compression and reduced clinical heterogeneity, in which baseline sepsis risk was uniformly elevated and discriminative ranking was inherently more difficult. In the ED, patient encounters represent a broad spectrum of illness severity, allowing the model to more effectively discriminate between those at low vs high risk of developing sepsis. In contrast, patients on inpatient wards and particularly in the ICU have a higher and more homogeneous baseline risk, with many exhibiting abnormal physiologic parameters or early organ dysfunction, compressing the range of predicted risk and potentially limiting achievable discrimination. These findings suggest that the model’s primary strength may lie in earlier phases of care in which timely identification of patients at risk may enable earlier intervention, while performance in higher-acuity settings is constrained by reduced residual uncertainty.

The choice of sepsis definition has major implications for model evaluation and clinical utility. While Sepsis-3 is often considered the most clinically contemporary definition, it was developed to optimize prognostic accuracy among patients with suspected infection rather than definitively identify true sepsis cases.^17^ As a result, its infection criteria are generally more sensitive but less specific than those of the ASE definition when compared with medical record review.^32^ In contrast, the ASE definition aims to improve specificity by focusing on patients with sustained antibiotic treatment and concurrent organ dysfunction, making it more suitable for surveillance and epidemiologic research.^33^ The SEP-1 definition, meanwhile, remains the mandated standard for hospital quality reporting in the US and is based on legacy, but still widely used, systemic inflammatory response syndrome criteria and organ dysfunction. Given their divergent purposes and patient populations, evaluating model performance across these definitions is essential for understanding generalizability and informing implementation in different clinical and policy contexts.

The vendor’s internal validation of the updated sepsis model, using the Sepsis-3 definition across the 3 training health care systems, reported an encounter-level AUROC of 0.84 to 0.88 compared with the AUROC of 0.89 observed in our study. The vendor’s findings showed a PPV ranging from 0.14 to 0.20 at a sensitivity threshold of 0.60, which is consistent with our observed PPV of 0.20. Unlike static models, such as version 1 of the sepsis model, that typically perform worse externally, version 2 of the sepsis model was locally trained on the health care system’s data, which may explain its comparable performance in our evaluation.^34^

Most prior studies have focused on the original sepsis model rather than the locally trained model.^12,14,35^ Wong et al^12^ reported an encounter-level AUROC of 0.63 for the original model using a SEP-1–based definition, with a sensitivity of 33% and PPV of 12% at a threshold score of 6. Cull et al^35^ found an AUROC of 0.83 with an *International Statistical Classification of Diseases, Tenth Revision* definition. More recently, Currey and Tarabichi^16^ showed that the updated sepsis model achieved an AUROC of 0.90 using Sepsis-3, outperforming the original model’s AUROC of 0.77. Although the vendor has reported wider use of the original sepsis model, transitioning to the updated model may improve predictive performance.

Building on Currey and Tarabichi’s^16^ evaluation of the updated sepsis model in a safety-net hospital ED, our study expands assessment across diverse clinical settings, including the ED, inpatient wards, and ICU, within a large health care system. Using the 3 sepsis definitions (Sepsis-3, SEP-1, and ASE) highlights the strengths and limitations of the model under varying use cases. We further assessed model timing compared with time zero definitions, incorporating both organ dysfunction and treatment, and quantified alert burden and false-positive rates, providing a more comprehensive evaluation of model’s utility for clinical decision support and quality improvement.

Kamran et al^10^ found that the original sepsis model’s performance degraded significantly from a baseline AUROC of 0.62 to an AUROC of 0.47 when restricted to predictions before sepsis treatment initiation using a composite outcome definition based on ASE and SEP-1 criteria. However, we found no significant degradation of model performance on any of the 3 sepsis outcome definitions when evaluated only on predictions prior to clinical recognition of sepsis.

Despite improved model performance compared with prior studies, the updated sepsis model still had relatively poor calibration and a high false-positive rate. Our decision curve analysis indicated that the model only provided net benefit at lower threshold probabilities in which the implication of false-positive rates was minimal. The model never achieved a PPV higher than 50%, even with high score thresholds that few patients ever reached. Decision support based on the model scores alone (at thresholds that favor sensitivity) may benefit the small proportion of patients who ultimately develop sepsis at the cost of increased alert burden and overtreatment for the majority of patients who do not.

Direct comparison of the updated sepsis model against other published sepsis predictive models using the Sepsis-3 outcome definition is challenged by differing patient populations with varying outcome incidence. The SepsisFinder model (Karolinska Institute) reported an outcome incidence of 9.7%, AUROC of 0.95, and AUPRC of 0.19.^36^ The DeepAISE model (Emory University) reported an AUROC of 0.90 in the ICU setting, with an outcome incidence of 5.6%.^37^ However, both models were evaluated on retrospective data only, and not after prospective implementation.

A model’s statistical performance during silent implementation does not necessarily equate to clinical benefit. While thousands of sepsis predictive models have been reported, relatively few studies have shown clinical impact, and only 1 study has shown a potential improvement in outcomes based on the model studied herein.^35,38,39,40^ In a before-and-after study, Cull et al^35^ reported a 44% decreased odds of sepsis-related in-hospital mortality after introduction of a clinical decision support alert based on the sepsis model. However, this study was limited by high potential for bias due to its reliance on billing codes to identify the sepsis cohort and the potential for increased detection of milder cases associated with heightened clinical awareness during quality improvement efforts.^41^ Further study of the clinical impact of the updated sepsis model is required.

Newer sepsis predictive models that leverage the capabilities of transformers and large language models to extract features from unstructured sources, such as physician notes and nursing documentation, have been reported.^42,43^ Unlike traditional machine learning models that rely primarily on structured data such as vital signs and laboratory results, large language models can extract clinically relevant information embedded in natural language, such as the suspicion of infection or the presence of altered mental status. Future versions of the sepsis model that incorporate unstructured data may improve performance.

### Limitations

This study had some limitations. We evaluated the sepsis model in a single health care system in the northeastern US. Other health care systems may achieve different model performance with differing patient demographics or disease burden. We mitigated this limitation by performing sensitivity analyses across differing clinical settings (community, academic, and critical access hospitals), as well as across demographic subgroups. The sepsis model studied herein is only 1 of many sepsis predictive models; its direct comparison against other models was outside the scope of this study. Finally, updated ASE outcome definitions have been submitted and are currently under consideration. Our analysis focused on the existing ASE definition, and not the updated definition.

## Conclusions

This diagnostic study of a locally trained early detection of sepsis model found moderate predictive performance, with model discrimination, precision, and lead time varying substantially by the sepsis definition applied. Although the model provided modest advance warning prior to sepsis onset, its high false-positive rate may limit clinical utility without careful threshold selection and targeted implementation strategies.

## Notes

Supplement 1.
eMethods.
**eTable 1.** Sepsis Outcome and Time Zero Definitions **eTable 2.** Description of Study Population **eTable 3.** Description of Study Population by Sepsis Outcome **eTable 4.** Detailed Decision Curve Data (Figure 1) **eTable 5.** Score Threshold Statistics (Figure 3) **eFigure 1.** Time From Hospital Arrival to Sepsis Time Zero **eFigure 2.** Model Performance Over Time **eFigure 3.** Prediction-Level Model Performance **eFigure 4.** Prediction-Level Model Performance in the Emergency Department **eFigure 5.** Prediction-Level Model Performance on Inpatient Wards **eFigure 6.** Prediction-Level Model Performance in the Intensive Care Unit **eFigure 7.** Classification Plots by Encounter Type **eFigure 8.** Encounter-Level Model Performance for Direct Hospital Admissions **eFigure 9.** Encounter-Level Model Performance for ED-to-Hospitalization Encounters **eFigure 10.** Encounter-Level Model Performance for OR-to-Hospitalization Encounters
eReferences.

Supplement 2.
Data Sharing Statement
