# Supplementary material for: Performance of a Sepsis Prediction Model Across Different Sepsis Definitions
Source: JAMA Netw Open. 2026 Apr 7;9(4):e265599. doi: 10.1001/jamanetworkopen.2026.5599 (PMC13058769; doi:10.1001/jamanetworkopen.2026.5599)
Supplement: Supplement 1. — eMethods. eTable 1. Sepsis Outcome and Time Zero Definitions eTable 2. Description of Study Population eTable 3. Description of Study Population by Sepsis Outcome eTable 4. Detailed Decision Curve Data (Figure 1) eTable 5. Score Threshold Statistics (Figure 3) eFigure 1. Time From Hospital Arrival to Sepsis Time Zero eFigure 2. Model Performance Over Time eFigure 3. Prediction-Level Model Performance eFigure 4. Prediction-Level Model Performance in the Emergency Department eFigure 5. Prediction-Level Model Performance on Inpatient Wards eFigure 6. Prediction-Level Model Performance in the Intensive Care Unit eFigure 7. Classification Plots by Encounter Type eFigure 8. Encounter-Level Model Performance for Direct Hospital Admissions eFigure 9. Encounter-Level Model Performance for ED-to-Hospitalization Encounters eFigure 10. Encounter-Level Model Performance for OR-to-Hospitalization Encounters eReferences. [file jamanetwopen-e265599-s001.pdf]

Supplemental Online Content

Dutta S, McMurry R, Tasi MC, et al. Performance of a sepsis prediction model across different sepsis definitions. *JAMA Netw Open*. 2026;9(4):e265599.
doi:10.1001/jamanetworkopen.2026.5599

eMethods

eTable 1. Sepsis Outcome and Time Zero Definitions

eTable 2. Description of Study Population

eTable 3. Description of Study Population by Sepsis Outcome

eTable 4. Detailed Decision Curve Data (Figure 1)

eTable 5. Score Threshold Statistics (Figure 3)

eFigure 1. Time From Hospital Arrival to Sepsis Time Zero

eFigure 2. Model Performance Over Time

eFigure 3. Prediction-Level Model Performance

eFigure 4. Prediction-Level Model Performance in the Emergency Department

eFigure 5. Prediction-Level Model Performance on Inpatient Wards

eFigure 6. Prediction-Level Model Performance in the Intensive Care Unit

eFigure 7. Classification Plots by Encounter Type

eFigure 8. Encounter-Level Model Performance for Direct Hospital Admissions

eFigure 9. Encounter-Level Model Performance for ED-to-Hospitalization Encounters

eFigure 10. Encounter-Level Model Performance for OR-to-Hospitalization Encounters

eReferences

This supplemental material has been provided by the authors to give readers additional information about their work.

© 2026 Dutta S et al. *JAMA Network Open*.

eMethods

Sample Size Calculation

To determine the necessary sample size, we employed the method outlined by Riley et al.[1] This involved iteratively calculating the AUROC confidence interval width across various candidate sample sizes. The goal was to identify the smallest sample size that would yield a confidence interval width of less than 0.05. We based this calculation on an assumed sepsis incidence of 2% within our healthcare system and a projected model AUROC of 0.8. Following this approach, we concluded that a minimum sample size of 15,333 encounters was required.

Outcome Definitions

The **Adult Sepsis Event (ASE)** algorithm was defined by a blood culture collection (regardless of result) and four consecutive days of antibiotics (or <4 days if administered through <=1 day prior to death, discharge to hospice or another acute care hospital, or transition to comfort measures) with concurrent organ dysfunction using binary thresholds adapted from the SOFA score ("eSOFA"). The first antibiotic day and organ dysfunction were required to occur within +/-2 calendar days of the blood culture draw. For this outcome, the sepsis time zero was defined as the earliest of blood culture collection, qualifying antibiotic administration, or organ dysfunction within the infection window.

The **CMS SEP-1** quality measure is a process-of-care bundle measure, not a purely clinical outcome definition. Official SEP-1 eligibility is determined using a complex abstraction algorithm that requires (1) specific ICD-10 septicemia, sepsis, severe sepsis, or septic shock diagnosis codes and (2) detailed chart-abstracted evidence of suspected infection, SIRS criteria, and organ dysfunction occurring within a 6-hour window. In addition, CMS SEP-1 explicitly excludes patients meeting numerous clinical criteria (e.g., comfort measures only, hospice, certain transfers, or early death) and relies on manual chart abstraction rather than computable rules.

In contrast, our SEP-1–based computable definition simplified SEP-1 into a computable clinical phenotype, requiring a sepsis ICD-10 diagnosis code, ≥2 SIRS criteria, and objective evidence of organ dysfunction within a fixed 6-hour window. Organ dysfunction included hypotension, initiation of mechanical ventilation, serum creatinine elevation (except if the patient was on dialysis), or elevations in bilirubin, platelets, or lactic acid, using thresholds that mirror the SEP-1 time zero definition. Unlike the CMS measure, our definition did not require documentation of suspected infection timing, bundle compliance elements (e.g., blood culture timing, antibiotic administration, fluid resuscitation), or application of SEP-1 exclusion criteria, and used laboratory and physiologic thresholds rather than chart-abstracted clinical intent. As a result, this definition approximated the clinical severity construct underlying SEP-1 but did not replicate the full CMS abstraction logic or its compliance-focused intent. Time zero for our definition was defined as the first time the patient met at least two SIRS criteria and had evidence of organ dysfunction.

© 2026 Dutta S et al. *JAMA Network Open*.

The **Sepsis-3** outcome was defined by the presence of culture collection, antibiotics, and increase of Sequential [Sepsis-related] Organ Failure Assessment (SOFA) score by 2 points. The time zero for this outcome was defined by the earliest of blood culture collection or antibiotic administration. The ESMv2 was trained by Epic on this outcome definition. we performed a sensitivity analysis of lead time for the Sepsis-3 outcome, evaluating the lead time using first qualifying antibiotic, first qualifying culture, and first SOFA increase as alternate time-zero definitions. We found that clinical suspicion (as indicated by getting cultures or giving antibiotics) preceded the increase in SOFA scores. Consequently, using SOFA score as the time zero definition for Sepsis-3 makes the model appear to provide more lead time, not less.

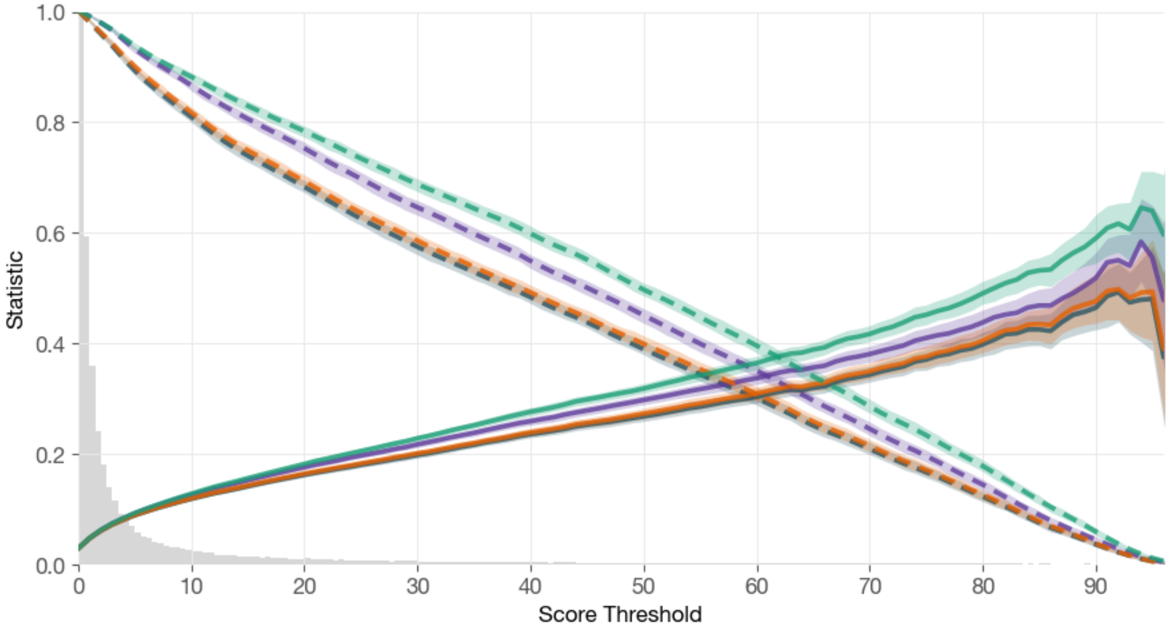

Sepsis-3	Recall	0.68	0.57	0.48	0.39	0.30	0.21	0.12
Sepsis-3	Precision	0.16	0.20	0.24	0.27	0.30	0.34	0.40
SOFA Increase	Recall	0.75	0.65	0.55	0.45	0.35	0.25	0.14
SOFA Increase	Precision	0.18	0.22	0.26	0.30	0.34	0.38	0.44
Culture	Recall	0.69	0.59	0.49	0.40	0.31	0.21	0.13
Culture	Precision	0.16	0.20	0.24	0.27	0.31	0.35	0.41
Antibiotics	Recall	0.78	0.69	0.60	0.50	0.40	0.29	0.18
Antibiotics	Precision	0.18	0.23	0.28	0.32	0.36	0.42	0.49

Classification plot for the ESMv2 model on the Sepsis-3 outcome using alternate time-zero definitions.

© 2026 Dutta S et al. *JAMA Network Open*.

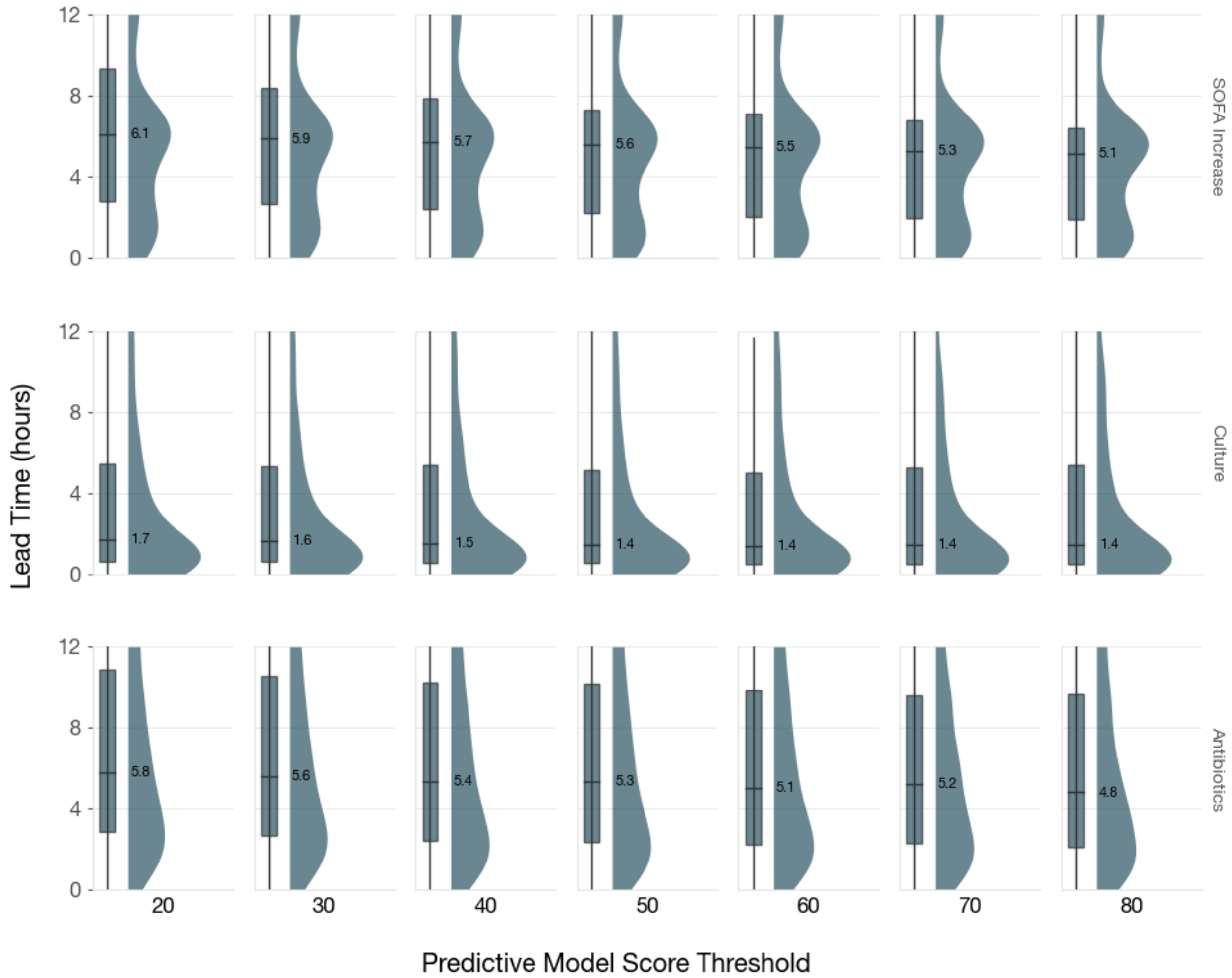

Lead time plot for the ESMv2 model on the Sepsis-3 outcome using alternate time-zero definitions.

Encounter and Prediction-Level Performance

The model performance was described at the encounter and prediction level. The encounter-level performance described model performance using the highest predictive model score prior to the outcome (sepsis time-zero) *or* hospital discharge if the outcome did not occur, while prediction-level performance determined if the outcome occurred within specific horizons of 8 hours and 24 hours after *each* prediction. Encounter-level performance can mask the fact that the outcome may occur shortly after the highest prediction without enough lead-time warning for clinical intervention, while prediction-level analysis can overemphasize patients with longer hospital stays due to the increased number of predictions. Recognizing the limitations of both prediction-level and encounter-level analyses, both were presented. For both methods, only predictions that occurred prior to the sepsis time-zero definition for each outcome definition were used.

Model performance was evaluated using the area under the receiver operator curve (AUROC), area under the precision recall curve (AUPRC), estimated calibration error (ECE), decision curve analysis, and

© 2026 Dutta S et al. *JAMA Network Open*.

lead-time analysis. The Receiver Operating Characteristic curve (ROC) assessed the model's ability to distinguish between positive and negative outcomes. It plotted the true-positive rate (sensitivity) against the false-positive rate (1 - specificity), where the AUROC of 0.5 indicated no discriminatory ability and 1.0 signified a perfect model. The AUPRC reflected the relationship between precision (positive predictive value) and recall (sensitivity). An AUPRC equivalent to the outcome's incidence suggests a random model that lacks discriminatory capacity. Given the typically low incidence of sepsis, the AUROC can present an overly optimistic view of performance, making the AUPRC a potentially more reliable metric for evaluating the model.[2] The ECE grouped predictions into decile bins according to their predicted probabilities, and then averaged the absolute difference between the average predicted probability and observed risk within each bin.[3] Decision curve analysis described the standardized net benefit relative to the threshold probability, a weighted metric that progressively penalizes false positives.[4,5]

We plotted ROC and precision-recall curves and computed the AUROC and AUPRC using bootstrapping to obtain 95% confidence intervals using resampling at the encounter level, and accounting for clustering at the patient level. The AUROC and AUPRC for the overall study population were calculated, as well as for population subgroups based on patient demographics, emergency severity index (ESI), encounter type, and hospital type. Calibration plots and decision curves were plotted both for the overall study population, as well as stratified by encounter type. The lead-time, i.e. the amount of time between when the model reached a specific score threshold and when the sepsis-time-zero definition was first met within the encounter, was plotted. AUROC and AUPRC was calculated by week to evaluate for temporal performance drift.

The model performance was reported at three specific pre-defined thresholds: High Recall (recall >= 0.80), Youden’s top left (the point on the ROC curve that maximizes the trade-off between sensitivity and specificity), and High Precision (precision >= 0.15). For each threshold we reported the precision, recall, false positive rate (FPR), number of encounters needed to evaluate (NNE) which represents the number of encounters the threshold was met for every encounter with the sepsis outcome, and the number and proportion of encounters in the overall study population that met that threshold.

© 2026 Dutta S et al. *JAMA Network Open*.

eTable 1. Sepsis Outcome and Time Zero Definitions

Outcome	Outcome Definition	Time-Zero Definition
ASE	Blood culture collection (regardless of result) and four consecutive days of antibiotics (or <4 days if administered through <=1 day prior to death, discharge to hospice or another acute care hospital, or transition to comfort measures) with concurrent organ dysfunction using binary thresholds adapted from the SOFA score ("eSOFA")	Earliest time of blood culture collection, qualifying antibiotic administration, or organ dysfunction within the +/- 2 day infection window (anchored to the blood culture collection day)
SEP-1	Sepsis ICD-10 diagnosis code for the encounter *and* at least two SIRS criteria *and* the presence of organ dysfunction within a 6-hour period. Organ dysfunction includes hypotension, initiation of mechanical ventilation, serum creatinine elevation (except if the patient was on dialysis), or elevations in bilirubin, platelets, or lactic acid	Earliest time when at least two SIRS criteria were met *with* concurrent organ dysfunction
Sepsis-3	Clinical culture collection (blood, urine, cerebrospinal fluid, etc.), parenteral antibiotic administration, and increase from the patient's baseline of Sequential [Sepsis-related] Organ Failure Assessment (SOFA) score by 2 points. The clinical culture must occur within 72 hours prior to or 24 hours after antibiotics administration. Increase in SOFA score must occur 48 hours prior to or 24 hours after time zero.	Earliest time of clinical culture collection or qualifying parenteral antibiotic administration.

Abbreviations: ASE, Adult Sepsis Event; SIRS, Systemic Inflammatory Response Syndrome

© 2026 Dutta S et al. *JAMA Network Open*.

eTable 2. Description of Study Population

Characteristic	Overall, N = 198,494	Academic Medical Center, N = 85,513	Community Hospital, N = 101,766	Critical Access Hospital, N = 11,215
**Age, median years (IQR)**	55 (36, 71)	54 (35, 69)	56 (36, 73)	54 (34, 72)
**Sex, n (%)**				
Female	108,763 (54.8%)	45,577 (53.3%)	57,362 (56.4%)	5,824 (51.9%)
Male	89,731 (45.2%)	39,936 (46.7%)	44,404 (43.6%)	5,391 (48.1%)
**Race, n (%)**				
Asian	6,940 (3.5%)	3,874 (4.5%)	2,930 (2.9%)	136 (1.2%)
Black or African American	23,709 (11.9%)	13,201 (15.4%)	9,361 (9.2%)	1,147 (10.2%)
Two or more races or Other	2,268 (1.1%)	996 (1.2%)	1,128 (1.1%)	144 (1.3%)
Unknown	27,495 (13.9%)	14,373 (16.8%)	11,623 (11.4%)	1,499 (13.4%)
White	138,082 (69.6%)	53,069 (62.1%)	76,724 (75.4%)	8,289 (73.9%)
**Ethnic Group, n (%)**				
Hispanic	31,810 (16.0%)	14,969 (17.5%)	15,838 (15.6%)	1,003 (8.9%)
Not Hispanic	159,669 (80.4%)	66,588 (77.9%)	83,477 (82.0%)	9,604 (85.6%)
Unknown	7,015 (3.5%)	3,956 (4.6%)	2,451 (2.4%)	608 (5.4%)
**Encounter Type, n (%)**				
Direct Hospital Admission	8,983 (4.5%)	7,558 (8.8%)	1,407 (1.4%)	18 (0.2%)
ED only, without admission	135,163 (68.1%)	51,988 (60.8%)	73,058 (71.8%)	10,117 (90.2%)
ED to Hospitalization	43,247 (21.8%)	17,563 (20.5%)	24,659 (24.2%)	1,025 (9.1%)
OB to Hospitalization	1,397 (0.7%)	856 (1.0%)	520 (0.5%)	21 (0.2%)
OR to Hospitalization	9,704 (4.9%)	7,548 (8.8%)	2,122 (2.1%)	34 (0.3%)

* Other includes American Indian or Alaskan Native and Native Hawaiian or Pacific Islander

Abbreviations: IQR, Interquartile Range; ED, Emergency Department; OB, Obstetrics; OR, Operating Room

© 2026 Dutta S et al. *JAMA Network Open*.

eTable 3. Description of Study Population by Sepsis Outcome

	SEP-1		ASE		Sepsis-3	
**Characteristic**	**Not Sepsis,** N = 196,128	**Sepsis,** N = 2,366	**Not Sepsis,** N = 194,613	**Sepsis,** N = 3,881	**Not Sepsis,** N = 192,662	**Sepsis,** N = 5,832
**Age, median years (IQR)**	55 (36, 71)	69 (58, 79)	55 (35, 71)	68 (58, 78)	55 (35, 71)	69 (57, 79)
**Sex, n (%)**						
Female	107,688 (54.9%)	1,075 (45.4%)	107,086 (55.0%)	1,677 (43.2%)	106,158 (55.1%)	2,605 (44.7%)
Male	88,440 (45.1%)	1,291 (54.6%)	87,527 (45.0%)	2,204 (56.8%)	86,504 (44.9%)	3,227 (55.3%)
**Hospital Type, n (%)**						
Academic Medical Center	84,336 (43.0%)	1,177 (49.7%)	83,086 (42.7%)	2,427 (62.5%)	82,159 (42.6%)	3,354 (57.5%)
Community Hospital	100,596 (51.3%)	1,170 (49.5%)	100,343 (51.6%)	1,423 (36.7%)	99,390 (51.6%)	2,376 (40.7%)
Critical Access Hospital	11,196 (5.7%)	19 (0.8%)	11,184 (5.7%)	31 (0.8%)	11,113 (5.8%)	102 (1.7%)
**Encounter Type, n (%)**						
Direct Hospital Admission	8,666 (4.4%)	317 (13.4%)	8,328 (4.3%)	655 (16.9%)	8,064 (4.2%)	919 (15.8%)
ED Only, Without Admission	135,119 (68.9%)	44 (1.9%)	135,157 (69.4%)	6 (0.2%)	134,789 (70.0%)	374 (6.4%)
ED to Hospitalization	41,297 (21.1%)	1,950 (82.4%)	40,227 (20.7%)	3,020 (77.8%)	38,919 (20.2%)	4,328 (74.2%)
OB to Hospitalization	1,397 (0.7%)	0 (0.0%)	1,396 (0.7%)	1 (0.0%)	1,396 (0.7%)	1 (0.0%)
OR to Hospitalization	9,649 (4.9%)	55 (2.3%)	9,505 (4.9%)	199 (5.1%)	9,494 (4.9%)	210 (3.6%)
**In-Hospital Mortality**, n (%)	1,940 (1.0%)	519 (21.9%)	1,732 (0.9%)	727 (18.7%)	1,630 (0.8%)	829 (14.2%)

Abbreviations: ED, Emergency Department; OB, Obstetrics; OR, Operating Room; ASE, Adult Sepsis Event

© 2026 Dutta S et al. *JAMA Network Open*.

eTable 4. Detailed Decision Curve Data (Figure 1)

	Threshold	Precision (%)	Recall (%)	FPR (%)	NNE	Lead Time (h)[a]	Encounters, No. (%)
**ASE**							
High recall	5	5.5 (5.3, 5.6)	81.8 (81.2, 83.2)	28.0 (28.0, 28.5)	18.2 (17.7, 18.8)	1.4 (0.5-14.5)	57,700 (29.1)
Youden top left	6	5.9 (5.7, 6.0)	79.3 (78.6, 80.7)	25.4 (25.4, 25.8)	17.1 (16.6, 17.7)	1.4 (0.5-14.8)	52,513 (26.5)
High precision	96	13.2 (3.8, 22.9)	0.1 (0.0, 0.3)	0.0 (0.0, 0.0)	7.6 (4.4, 26.2)	1.1 (0.5-2.1)	38 (0)
**SEP-1**							
High recall	26	9.4 (9.1, 9.9)	80.4 (79.6, 81.6)	9.4 (9.2, 9.6)	10.7 (10.1, 11.0)	4.2 (1.2-20.6)	20,285 (10.2)
Youden top left	15	6.8 (6.6, 7.1 9.9)	89.1 (88.1, 90.2)	14.8 (14.6, 14.9)	14.7 (14.0, 15.2)	4.5 (1.3-23.1)	31,045 (15.6)
High precision	52	15.2 (14.7, 16.0 9.9)	55.5 (53.8, 57.4)	3.7 (3.6, 3.9)	6.6 (6.2, 6.8)	3.3 (0.9-16.7)	8,668 (4.4)
**Sepsis-3**							
High recall	10	11.9 (11.7, 12.3)	80.8 (80.3, 82.5)	18.0 (17.9, 18.3)	8.4 (8.1, 8.6)	3.4 (0.9-22.3)	39,459 (19.9)
Youden top left	9PP	11.4 (11.1, 11.7)	82.4 (81.8, 83.8)	19.3 (19.2, 19.6)	8.7 (8.5, 9.0)	3.4 (0.9-22.4)	42,032 (21.2)
High precision	17	15.1 (14.7, 15.6)	71.6 (70.6, 73.0)	12.2 (12.1, 12.4)	6.6 (6.4, 6.8)	3.3 (0.8-22.4)	27,670 (13.9)

Abbreviations: FPR, false-positive rate; NNE, number of encounters needed to detect 1 true-positive encounter

[a]The median (IQR) lead time represents the time between when the model first reached the defined threshold and the first occurrence of time 0 for the sepsis outcome definition. The number and proportion of total encounters that reached the defined threshold

© 2026 Dutta S et al. *JAMA Network Open*.

eTable 5. Score Threshold Statistics (Figure 3)

	Score Threshold								
	**10**	**20**	**30**	**40**	**50**	**60**	**70**	**80**	**90**
**ASE**									
Recall	0.72	0.58	0.48	0.39	0.31	0.24	0.16	0.08	0.02
Precision	0.07	0.09	0.11	0.13	0.14	0.16	0.18	0.19	0.19
**SEP-1**									
Recall	0.93	0.86	0.77	0.68	0.58	0.47	0.34	0.20	0.06
Precision	0.05	0.08	0.10	0.13	0.15	0.17	0.19	0.22	0.25
**Sepsis-3**									
Recall	0.81	0.68	0.57	0.48	0.39	0.30	0.21	0.12	0.04
Precision	0.12	0.16	0.20	0.24	0.27	0.30	0.34	0.40	0.47

© 2026 Dutta S et al. *JAMA Network Open*.

eFigure 1. Time From Hospital Arrival to Sepsis Time Zero

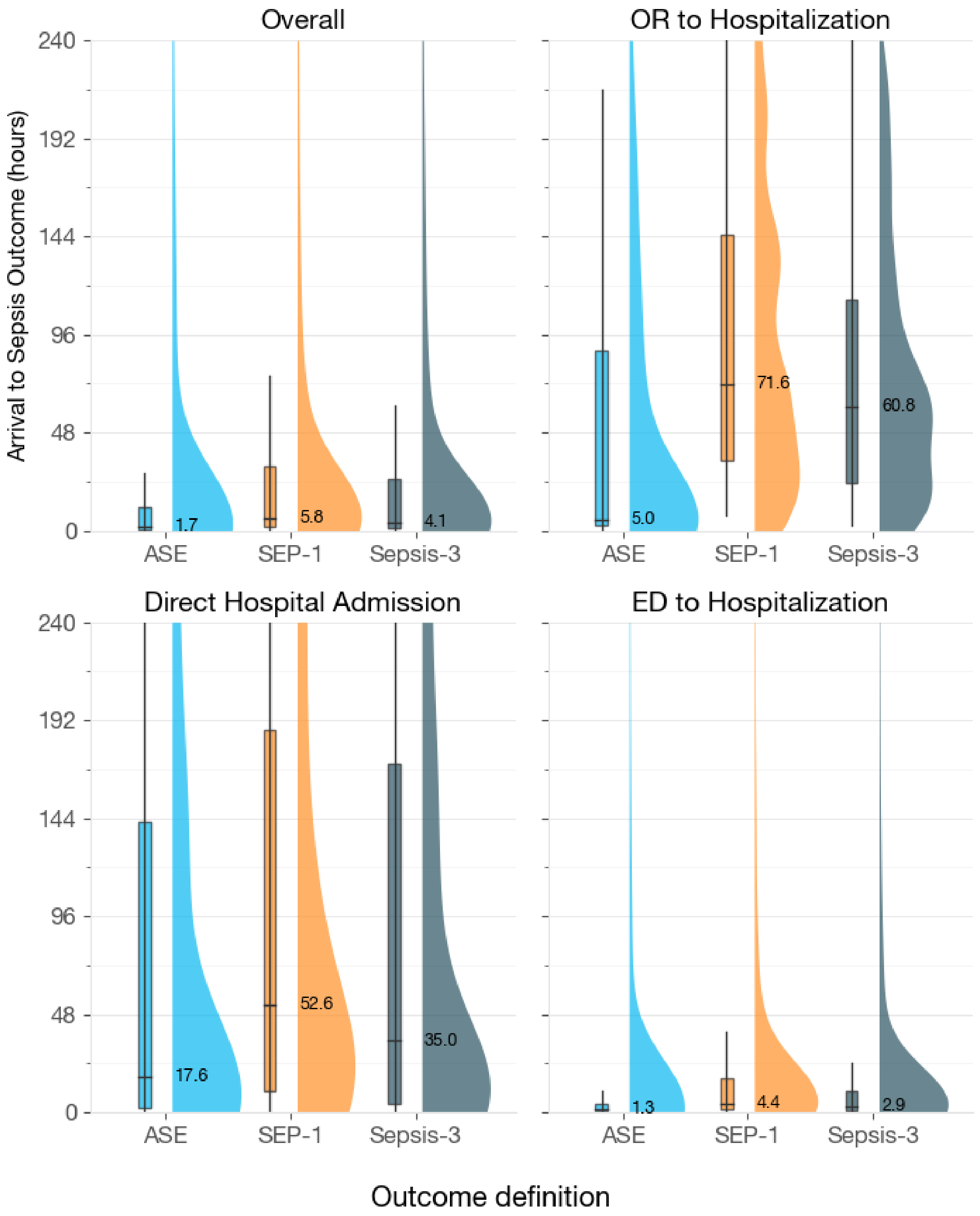

This boxplot depicts the time between hospital arrival to the first time the patient meets the sepsis time-zero definition, per sepsis outcome.

Abbreviations: ASE, Adult Sepsis Event; ED, Emergency Department; OR, Operating Room

© 2026 Dutta S et al. *JAMA Network Open*.

eFigure 2. Model Performance Over Time

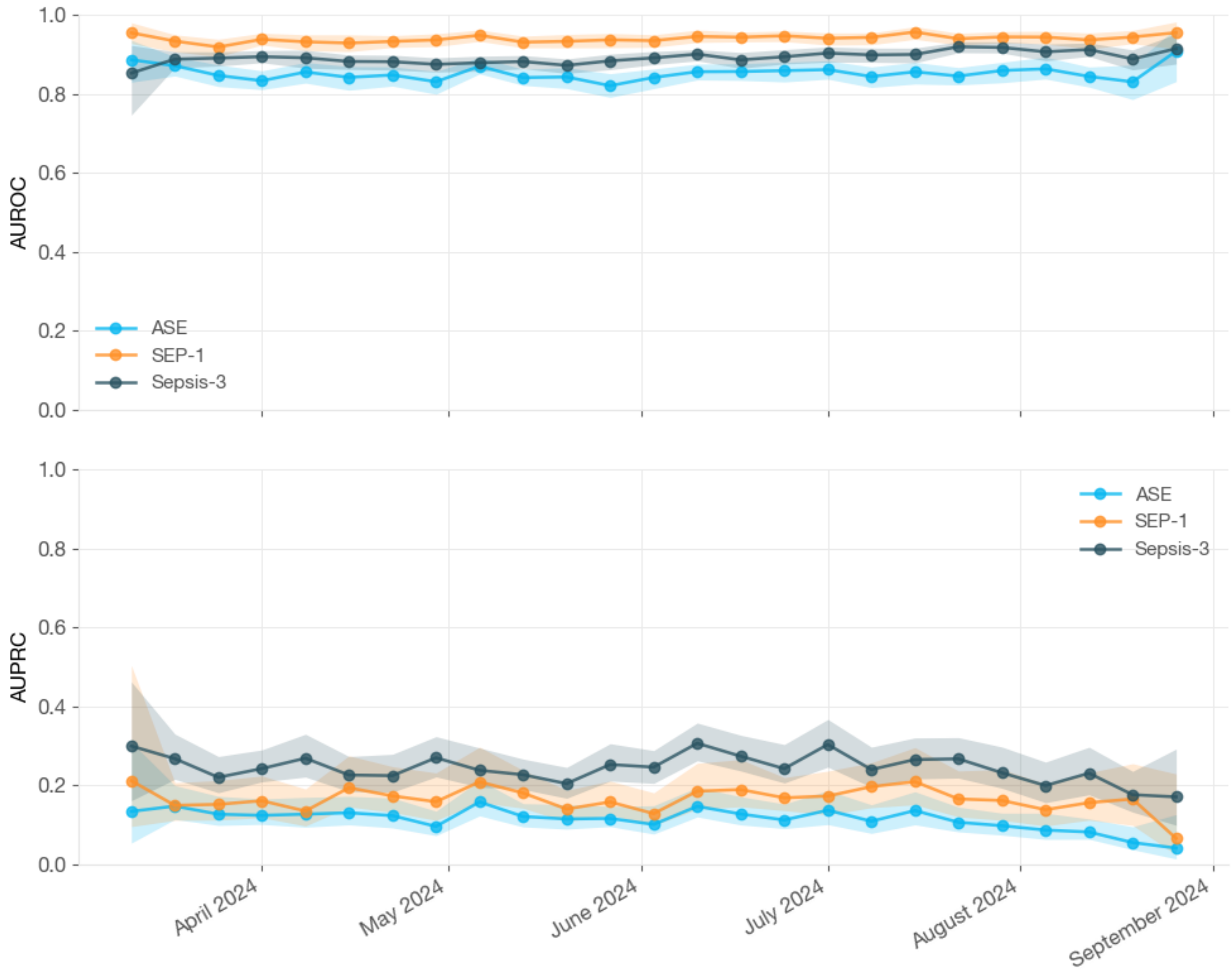

Weekly AUROC (top) and AUPRC (bottom) are shown for the ESMv2 model evaluated against the three sepsis definitions. Shaded bands represent 95% confidence intervals around point estimates.

© 2026 Dutta S et al. *JAMA Network Open*.

eFigure 3. Prediction-Level Model Performance

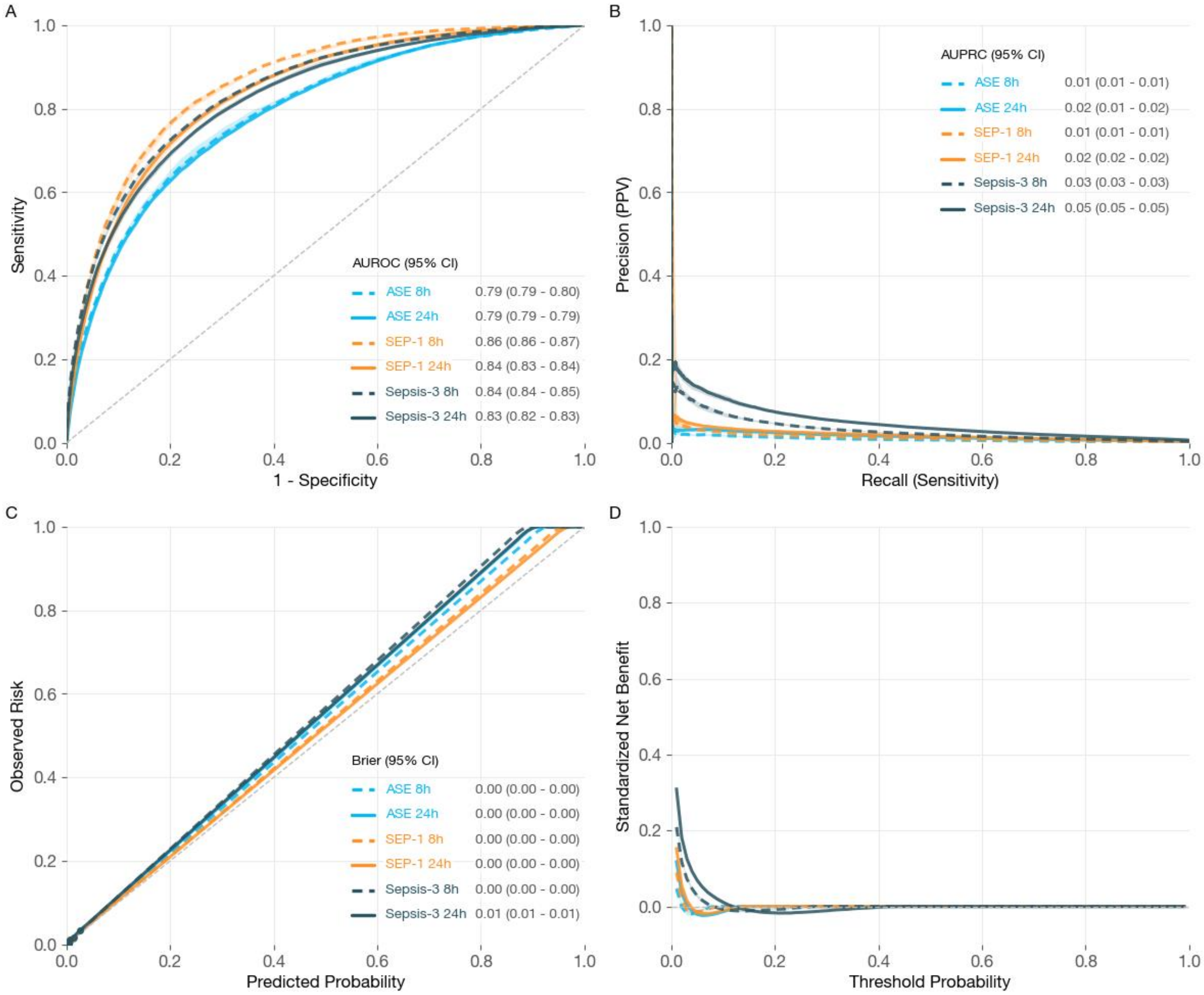

The ability of the ESMv2 to predict the three sepsis outcomes at the prediction level. Each color represents a different sepsis outcome. Panel A: Receiver operating characteristic curve. The diagonal dashed line represents a random model. Panel B: Precision-recall curve. The dashed horizontal-colored lines represent the incidence per sepsis outcome. Panel C: Model Calibration. The diagonal dashed line represents perfect calibration. Panel D: Decision Curves.

Abbreviations: AUROC, area under the receiver operating characteristic curve; AUPRC, area under the precision recall curve; ECE, estimated calibration error; PPV, positive predictive value; ASE, Adult Sepsis Event.

© 2026 Dutta S et al. *JAMA Network Open*.

eFigure 4. Prediction-Level Model Performance in the Emergency Department

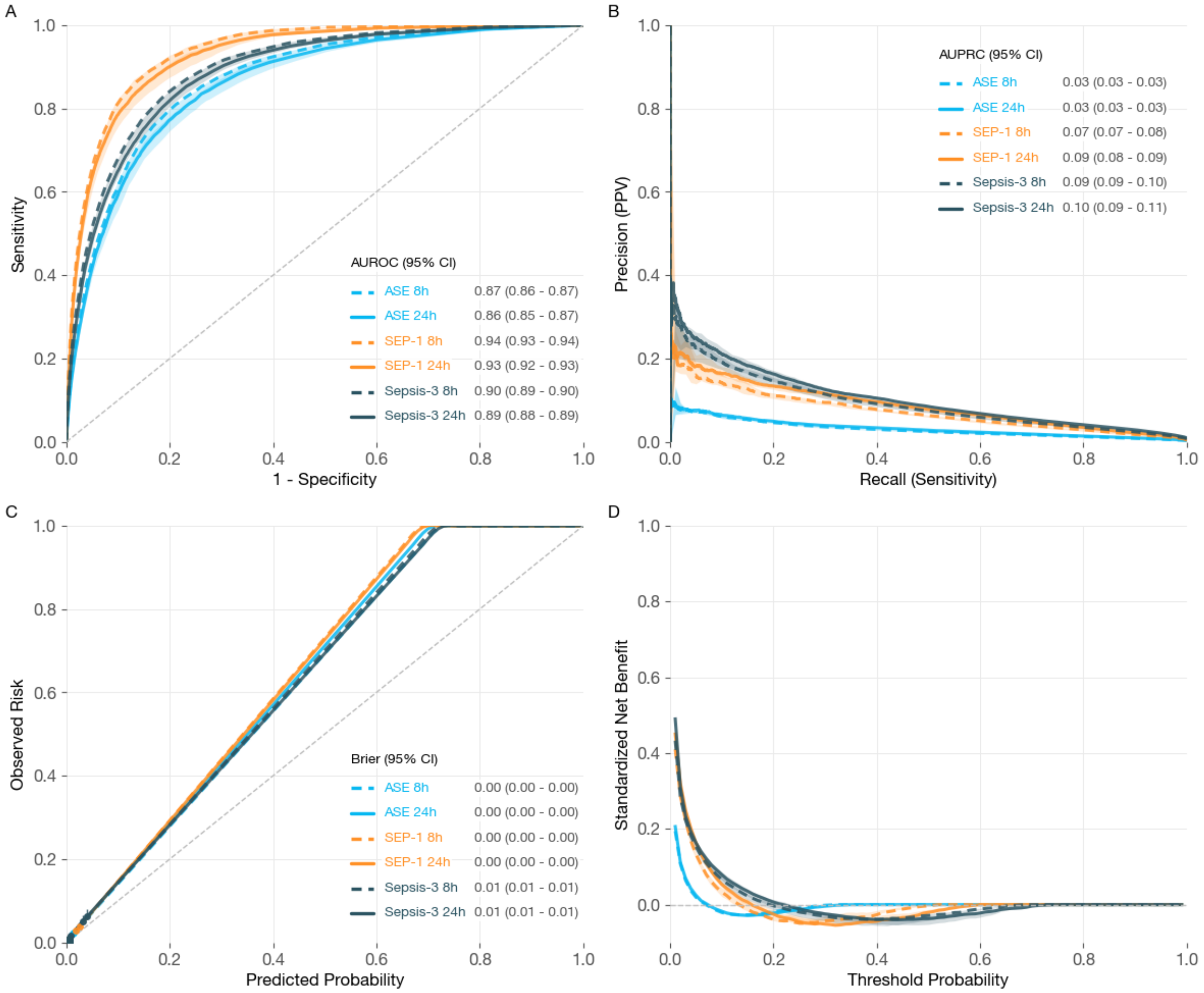

The ability of the ESMv2 to predict the three sepsis outcomes at the prediction level for predictions made in the emergency department. Each color represents a different sepsis outcome. Panel A: Receiver operating characteristic curve. The diagonal dashed line represents a random model. Panel B: Precision-recall curve. The dashed horizontal-colored lines represent the incidence per sepsis outcome. Panel C: Model Calibration. The diagonal dashed line represents perfect calibration. Panel D: Decision Curves.

Abbreviations: AUROC, area under the receiver operating characteristic curve; AUPRC, area under the precision recall curve; PPV, positive predictive value; ASE, Adult Sepsis Event.

© 2026 Dutta S et al. *JAMA Network Open*.

eFigure 5. Prediction-Level Model Performance on Inpatient Wards

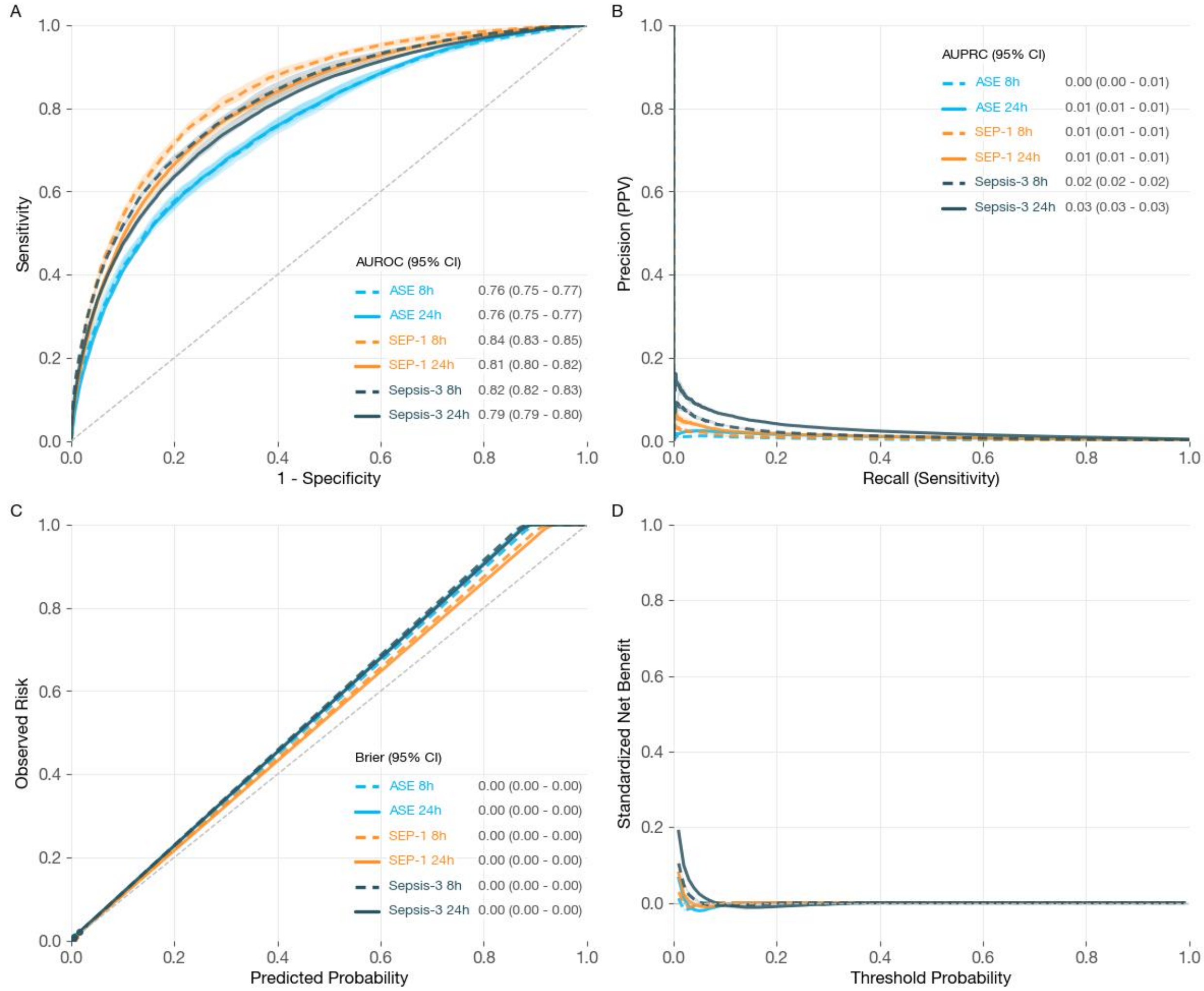

The ability of the ESMv2 to predict the three sepsis outcomes at the prediction level for predictions made on inpatient wards. Each color represents a different sepsis outcome. Panel A: Receiver operating characteristic curve. The diagonal dashed line represents a random model. Panel B: Precision-recall curve. The dashed horizontal-colored lines represent the incidence per sepsis outcome. Panel C: Model Calibration. The diagonal dashed line represents perfect calibration. Panel D: Decision Curves.

Abbreviations: AUROC, area under the receiver operating characteristic curve; AUPRC, area under the precision recall curve; PPV, positive predictive value; ASE, Adult Sepsis Event.

© 2026 Dutta S et al. *JAMA Network Open*.

eFigure 6. Prediction-Level Model Performance in the Intensive Care Unit

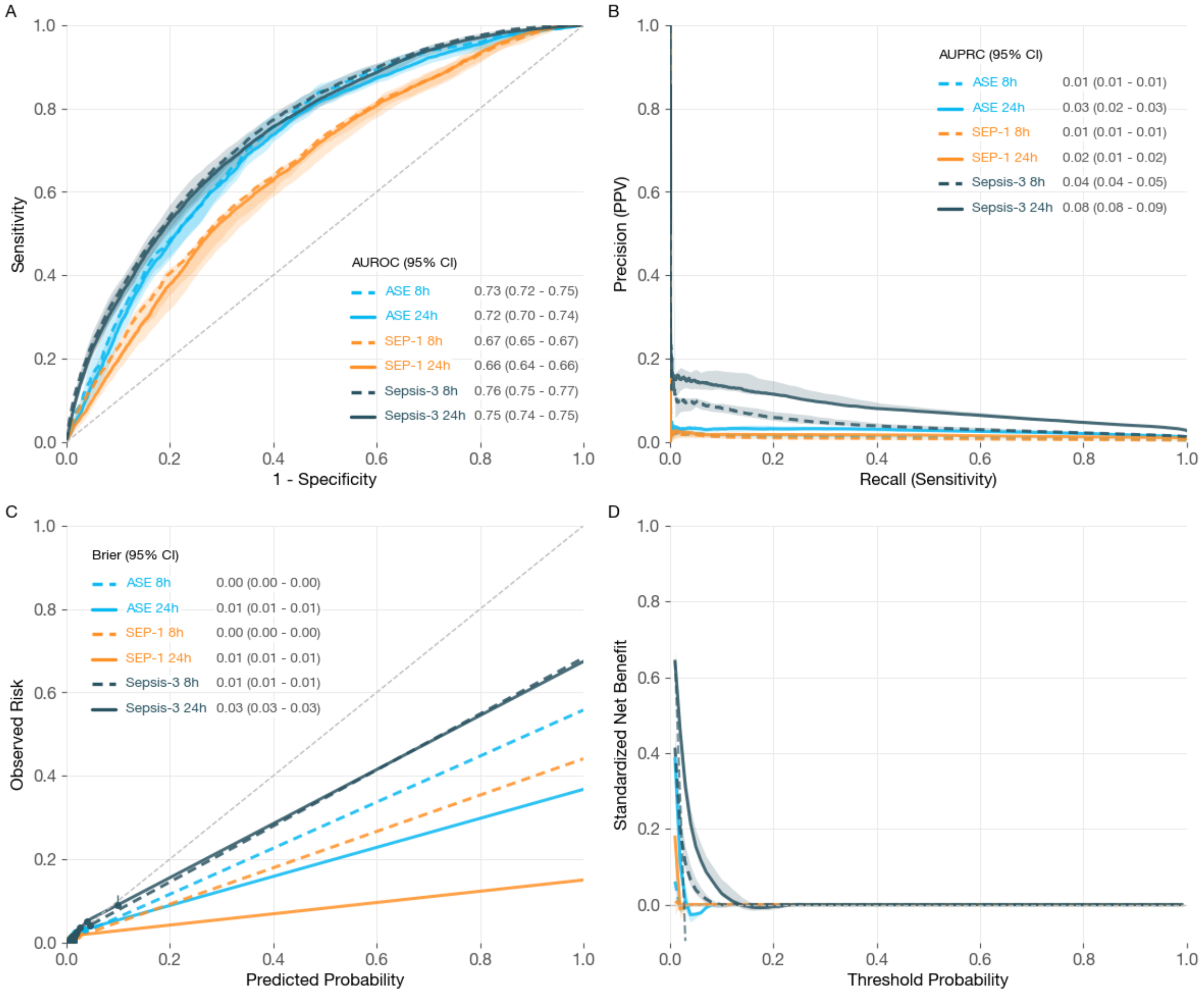

The ability of the ESMv2 to predict the three sepsis outcomes at the prediction level for predictions made in the intensive care unit. Each color represents a different sepsis outcome. Panel A: Receiver operating characteristic curve. The diagonal dashed line represents a random model. Panel B: Precision-recall curve. The dashed horizontal-colored lines represent the incidence per sepsis outcome. Panel C: Model Calibration. The diagonal dashed line represents perfect calibration. Panel D: Decision Curves.

Abbreviations: AUROC, area under the receiver operating characteristic curve; AUPRC, area under the precision recall curve; PPV, positive predictive value; ASE, Adult Sepsis Event.

© 2026 Dutta S et al. *JAMA Network Open*.

eFigure 7. Classification Plots by Encounter Type

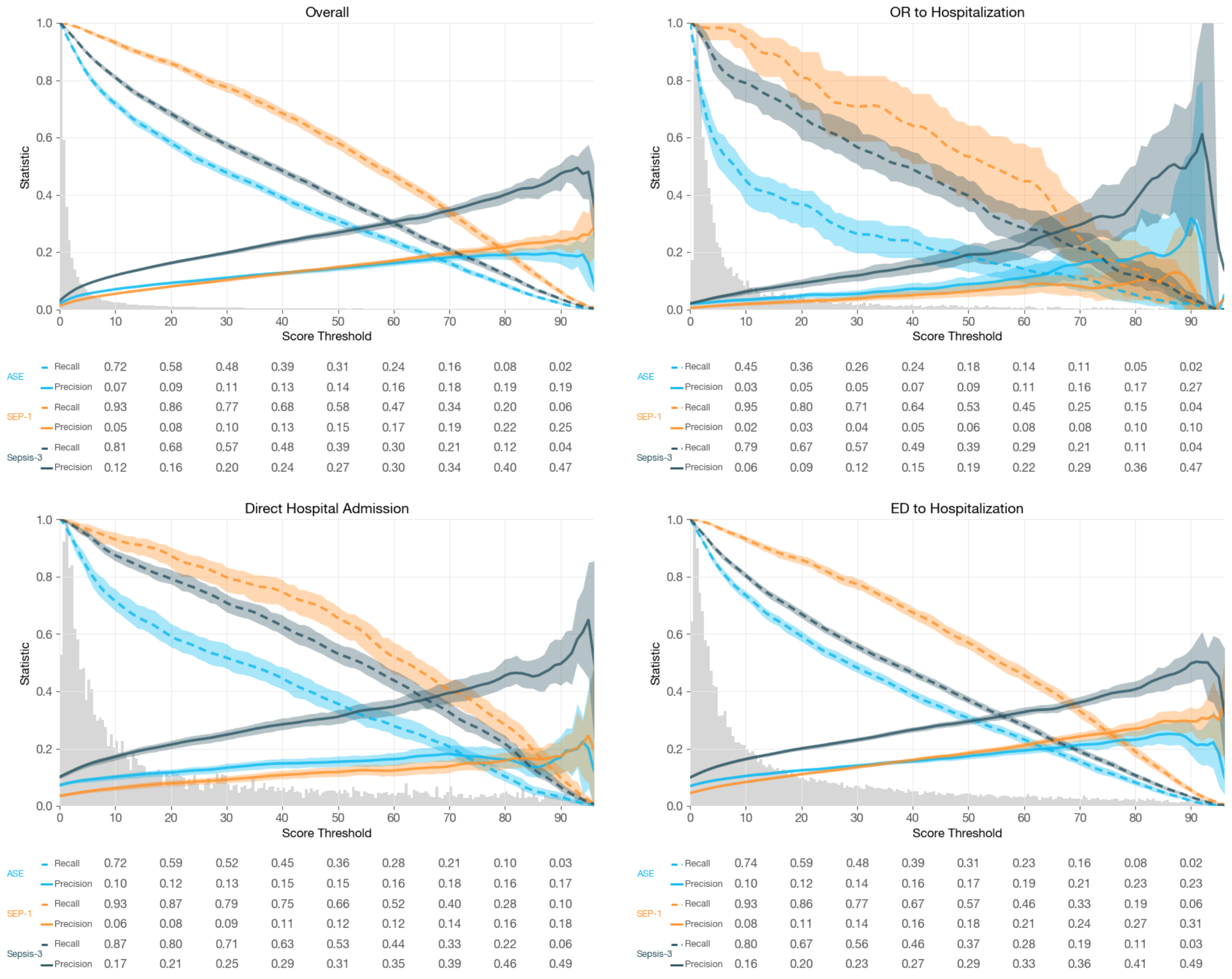

Overall

ASE	Recall	0.72	0.58	0.48	0.39	0.31	0.24	0.16	0.08	0.02
	Precision	0.07	0.09	0.11	0.13	0.14	0.16	0.18	0.19	0.19
SEP-1	Recall	0.93	0.86	0.77	0.68	0.58	0.47	0.34	0.20	0.06
	Precision	0.05	0.08	0.10	0.13	0.15	0.17	0.19	0.22	0.25
Sepsis-3	Recall	0.81	0.68	0.57	0.48	0.39	0.30	0.21	0.12	0.04
	Precision	0.12	0.16	0.20	0.24	0.27	0.30	0.34	0.40	0.47

OR to Hospitalization

ASE	Recall	0.45	0.36	0.26	0.24	0.18	0.14	0.11	0.05	0.02
	Precision	0.03	0.05	0.05	0.07	0.09	0.11	0.16	0.17	0.27
SEP-1	Recall	0.95	0.80	0.71	0.64	0.53	0.45	0.25	0.15	0.04
	Precision	0.02	0.03	0.04	0.05	0.06	0.08	0.08	0.10	0.10
Sepsis-3	Recall	0.79	0.67	0.57	0.49	0.39	0.29	0.21	0.11	0.04
	Precision	0.06	0.09	0.12	0.15	0.19	0.22	0.29	0.36	0.47

Direct Hospital Admission

ASE	Recall	0.72	0.59	0.52	0.45	0.36	0.28	0.21	0.10	0.03
	Precision	0.10	0.12	0.13	0.15	0.15	0.16	0.18	0.16	0.17
SEP-1	Recall	0.93	0.87	0.79	0.75	0.66	0.52	0.40	0.28	0.10
	Precision	0.06	0.08	0.09	0.11	0.12	0.12	0.14	0.16	0.18
Sepsis-3	Recall	0.87	0.80	0.71	0.63	0.53	0.44	0.33	0.22	0.06
	Precision	0.17	0.21	0.25	0.29	0.31	0.35	0.39	0.46	0.49

ED to Hospitalization

ASE	Recall	0.74	0.59	0.48	0.39	0.31	0.23	0.16	0.08	0.02
	Precision	0.10	0.12	0.14	0.16	0.17	0.19	0.21	0.23	0.23
SEP-1	Recall	0.93	0.86	0.77	0.67	0.57	0.46	0.33	0.19	0.06
	Precision	0.08	0.11	0.14	0.16	0.18	0.21	0.24	0.27	0.31
Sepsis-3	Recall	0.80	0.67	0.56	0.46	0.37	0.28	0.19	0.11	0.03
	Precision	0.16	0.20	0.23	0.27	0.29	0.33	0.36	0.41	0.49

The test characteristics of the ESMv2 predictive model were plotted by the highest predictive model score within the encounter (prior to the sepsis outcome time-zero or hospital discharge), stratified by encounter type. The recall (or sensitivity) is represented by dashed lines, and the precision (or positive predictive value) is represented as solid lines. Higher thresholds provide greater precision but less recall. The gray background represents a histogram of the maximum predictive model scores per patient encounter.

Abbreviations: ASE, Adult Sepsis Event

© 2026 Dutta S et al. *JAMA Network Open*.

eFigure 8. Encounter-level Model Performance for Direct Hospital Admissions

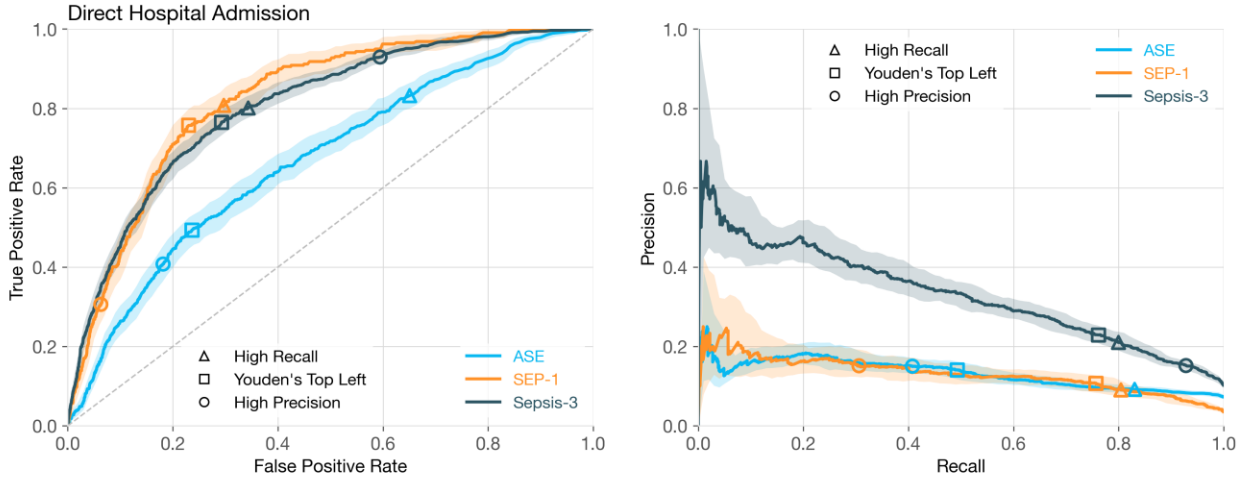

	Threshold	Precision (%)	Recall (%)	FPR (%)	NNE	Lead Time (hrs)	Encounters (n, %)
ASE							
△ High Recall	5	9.2 (8.4, 9.8)	83.1 (80.1, 86.4)	64.6 (64.0, 66.3)	10.9 (10.2, 11.9)	21.2 (1.5-140.4)	5,922 (65.9%)
□ Youden's Top Left	34	14.1 (12.8, 15.6)	49.3 (45.6, 53.3)	23.6 (22.7, 24.5)	7.1 (6.4, 7.8)	29.4 (1.3-139.5)	2,291 (25.5%)
○ High Precision	44	15.0 (13.5, 16.7)	40.8 (37.0, 45.1)	18.1 (17.3, 19.0)	6.7 (6.0, 7.4)	25.0 (1.5-139.9)	1,777 (19.8%)
SEP-1							
△ High Recall	29	9.0 (8.0, 10.2)	80.4 (76.3, 84.9)	29.6 (28.6, 30.7)	11.1 (9.8, 12.5)	31.4 (7.1-137.5)	2,822 (31.4%)
□ Youden's Top Left	39	10.7 (9.4, 12.2)	75.7 (70.7, 79.9)	23.0 (22.1, 24.0)	9.3 (8.2, 10.7)	28.6 (5.5-122.5)	2,235 (24.9%)
○ High Precision	78	15.1 (12.2, 17.7)	30.6 (26.1, 35.0)	6.3 (5.8, 6.8)	6.6 (5.6, 8.2)	27.0 (3.9-119.8)	641 (7.1%)
Sepsis-3							
△ High Recall	19	21.0 (19.7, 22.2)	80.0 (77.8, 83.2)	34.3 (33.3, 35.5)	4.8 (4.5, 5.1)	26.4 (3.7-131.5)	3,499 (39.0%)
□ Youden's Top Left	24	22.9 (21.4, 24.2)	76.2 (73.7, 79.6)	29.2 (28.2, 30.3)	4.4 (4.1, 4.7)	24.0 (3.6-120.2)	3,057 (34.0%)
○ High Precision	6	15.2 (14.1, 16.0)	92.8 (91.4, 94.3)	59.1 (58.4, 60.6)	6.6 (6.3, 7.1)	29.1 (3.3-153.6)	5,618 (62.5%)

Precision, recall, false positive rate (FPR), lead time before sepsis time-zero, and the number of encounters needing evaluation to identify one true positive in the direct hospital admissions study population at three pre-specified threshold points. High Recall identifies the threshold to achieve recall of 0.80. Youden's Top Left is the threshold that best balances precision and recall. High Precision identifies the model threshold needed to achieve a PPV of 0.15. The False Positive Rate (FPR) and Number of Encounters needed to Evaluate (NNE) to detect one true positive is reported. The Encounters (n, %) displays the number and proportion of total encounters that met the specified threshold within the encounter.

eFigure 9. Encounter-level Model Performance for ED-to-Hospitalization Encounters

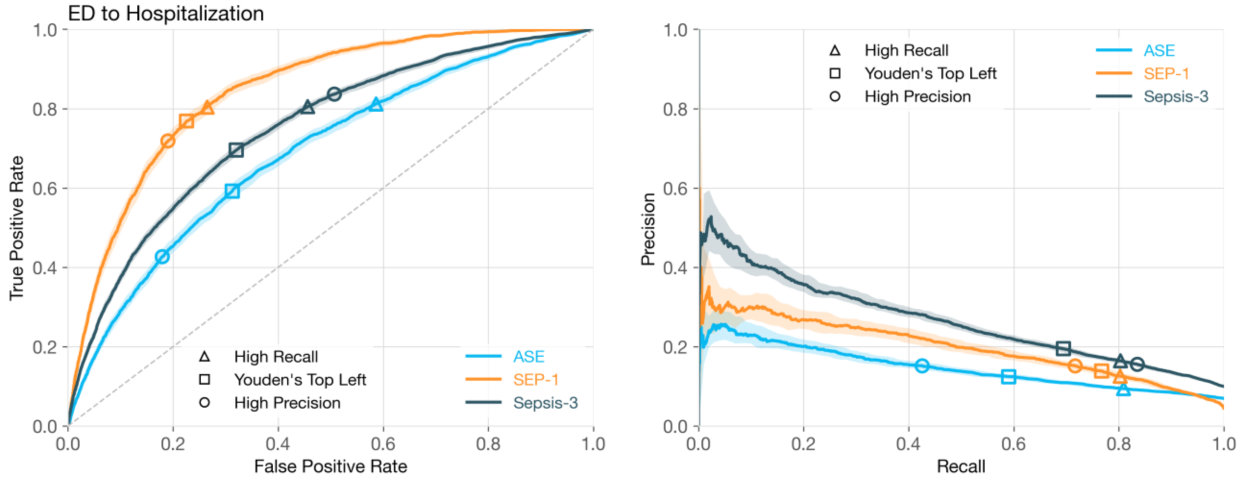

	Threshold	Precision (%)	Recall (%)	FPR (%)	NNE	Lead Time (hrs)	Encounters (n, %)
ASE							
△ High Recall	6	9.4 (9.0, 9.8)	80.9 (79.9, 82.5)	58.2 (58.0, 59.1)	10.6 (10.2, 11.1)	1.0 (0.4-4.5)	25,872 (59.8%)
□ Youden's Top Left	20	12.5 (12.0, 13.0)	59.0 (57.4, 61.1)	31.1 (30.7, 31.8)	8.0 (7.7, 8.3)	0.9 (0.4-4.8)	14,312 (33.1%)
○ High Precision	36	15.2 (14.5, 15.9)	42.6 (41.2, 44.6)	17.9 (17.5, 18.3)	6.6 (6.3, 6.9)	0.8 (0.3-4.1)	8,478 (19.6%)
SEP-1							
△ High Recall	26	12.6 (12.0, 13.1)	80.3 (78.7, 82.1)	26.4 (26.0, 26.9)	8.0 (7.6, 8.3)	3.2 (1.1-12.0)	12,469 (28.8%)
□ Youden's Top Left	31	13.9 (13.3, 14.4)	76.7 (75.1, 78.9)	22.5 (22.2, 23.0)	7.2 (6.9, 7.5)	2.9 (1.0-11.5)	10,794 (25.0%)
○ High Precision	36	15.2 (14.5, 15.8)	71.7 (70.0, 73.7)	18.9 (18.6, 19.4)	6.6 (6.3, 6.9)	2.8 (1.0-11.1)	9,223 (21.3%)
Sepsis-3							
△ High Recall	10	16.4 (15.9, 16.9)	80.3 (79.4, 81.5)	45.4 (45.0, 46.1)	6.1 (5.9, 6.3)	2.4 (0.7-9.2)	21,148 (48.9%)
□ Youden's Top Left	18	19.5 (18.8, 20.0)	69.4 (68.1, 70.8)	31.9 (31.5, 32.5)	5.1 (5.0, 5.3)	2.2 (0.7-9.0)	15,419 (35.7%)
○ High Precision	8	15.6 (15.0, 15.9)	83.5 (82.5, 84.5)	50.4 (50.1, 51.2)	6.4 (6.3, 6.7)	2.5 (0.8-9.8)	23,244 (53.7%)

Precision, recall, false positive rate (FPR), lead time before sepsis time-zero, and the number of encounters needing evaluation to identify one true positive in the ED to Hospitalization study population at three pre-specified threshold points. High Recall identifies the threshold to achieve recall of 0.80. Youden's Top Left is the threshold that best balances precision and recall. High Precision identifies the model threshold needed to achieve a PPV of 0.15. The False Positive Rate (FPR) and Number of Encounters needed to Evaluate (NNE) to detect one true positive is reported. The Encounters (n, %) displays the number and proportion of total encounters that met the specified threshold within the encounter.

© 2026 Dutta S et al. *JAMA Network Open*.

eFigure 10. Encounter-level Model Performance for OR-to-Hospitalization Encounters

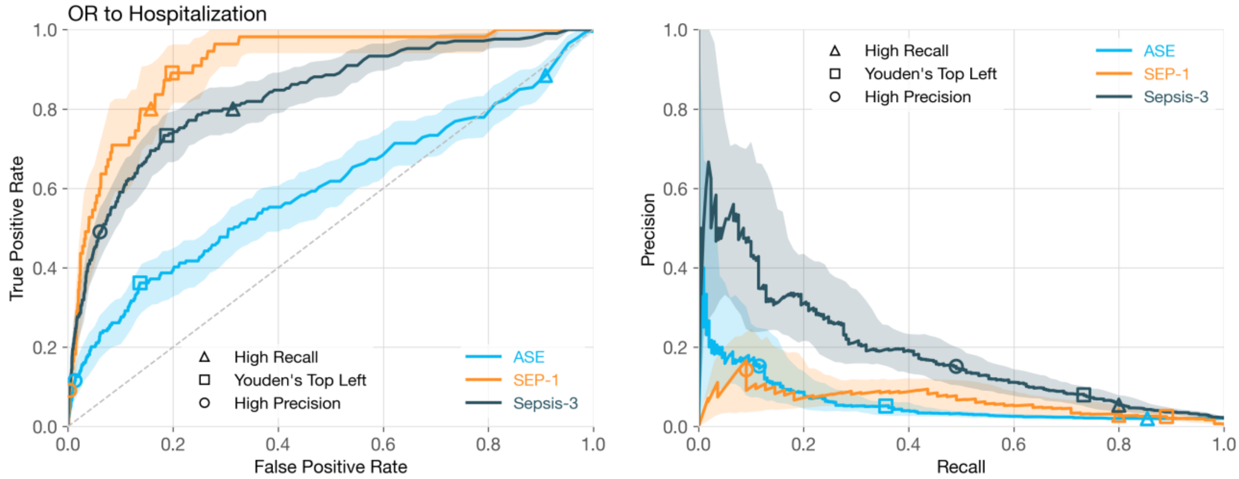

	Threshold	Precision (%)	Recall (%)	FPR (%)	NNE	Lead Time (hrs)	Encounters (n, %)
ASE							
△ High Recall	1	2.0 (1.7, 2.3)	85.4 (84.9, 92.3)	88.4 (90.3, 91.5)	50.4 (43.2, 58.1)	9.1 (2.7-103.5)	8,575 (88.4%)
□ Youden's Top Left	22	5.2 (4.1, 6.3)	35.7 (28.8, 41.6)	13.6 (13.0, 14.5)	19.2 (15.8, 24.5)	68.9 (11.3-128.0)	1,366 (14.1%)
○ High Precision	68	15.2 (10.4, 21.3)	11.6 (7.5, 16.0)	1.3 (1.1, 1.5)	6.6 (4.7, 9.7)	69.9 (29.2-136.0)	151 (1.6%)
SEP-1							
△ High Recall	20	2.8 (2.0, 3.6)	80.0 (71.0, 90.9)	15.8 (15.2, 16.6)	35.6 (28.1, 49.6)	28.0 (7.2-114.7)	1,568 (16.2%)
□ Youden's Top Left	16	2.5 (1.8, 3.2)	89.1 (81.6, 96.3)	19.8 (19.0, 20.6)	39.9 (31.4, 54.9)	31.3 (8.3-118.0)	1,956 (20.2%)
○ High Precision	87	14.3 (3.1, 25.9)	9.1 (1.8, 16.7)	0.3 (0.2, 0.4)	7.0 (3.9, 32.0)	3.4 (2.2-7.1)	35 (0.4%)
Sepsis-3							
△ High Recall	8	5.4 (4.5, 6.1)	80.0 (73.9, 85.6)	31.2 (30.6, 32.4)	18.6 (16.3, 22.2)	52.0 (19.7-111.5)	3,128 (32.2%)
□ Youden's Top Left	16	8.0 (6.7, 9.2)	73.3 (66.1, 79.1)	18.7 (17.9, 19.6)	12.5 (10.9, 14.8)	54.9 (12.8-109.9)	1,932 (19.9%)
○ High Precision	40	15.1 (12.4, 17.8)	49.0 (42.8, 55.0)	6.1 (5.6, 6.5)	6.6 (5.6, 8.1)	49.4 (11.8-98.3)	680 (7.0%)

Precision, recall, false positive rate (FPR), lead time before sepsis time-zero, and the number of encounters needing evaluation to identify one true positive in the OR to Hospitalization study population at three pre-specified threshold points. High Recall identifies the threshold to achieve recall of 0.80. Youden's Top Left is the threshold that best balances precision and recall. High Precision identifies the model threshold needed to achieve a PPV of 0.15. The False Positive Rate (FPR) and Number of Encounters needed to Evaluate (NNE) to detect one true positive is reported. The Encounters (n, %) displays the number and proportion of total encounters that met the specified threshold within the encounter.

© 2026 Dutta S et al. *JAMA Network Open*.

eReferences

1. Riley RD, Debray TPA, Collins GS, et al. Minimum sample size for external validation of a clinical prediction model with a binary outcome. *Stat Med*. 2021;40(19):4230-4251. doi:10.1002/sim.9025

2. Saito T, Rehmsmeier M. The Precision-Recall plot is more informative than the ROC plot when evaluating binary classifiers on imbalanced datasets. *PLOS ONE*. 2015;10(3):1-21. doi:10.1371/journal.pone.0118432

3. Huang Y, Li W, Macheret F, Gabriel RA, Ohno-Machado L. A tutorial on calibration measurements and calibration models for clinical prediction models. *J Am Med Inform Assoc*. 2020;27(4):621-633. doi:10.1093/jamia/ocz228

4. Vickers AJ, Calster B van, Steyerberg EW. A simple, step-by-step guide to interpreting decision curve analysis. *Diagn Progn Res*. 2019;3(1). doi:10.1186/s41512-019-0064-7

5. Sadatsafavi M, Adibi A, Puhan M, Gershon A, Aaron SD, Sin DD. Moving beyond AUC: decision curve analysis for quantifying net benefit of risk prediction models. *Eur Respir J*. 2021;58(5).

© 2026 Dutta S et al. *JAMA Network Open*.
