# Supplementary material for: Performance of a Sepsis Prediction Model Across Different Sepsis Definitions
Source: JAMA Netw Open. 2026 Apr 7;9(4):e265599. doi: 10.1001/jamanetworkopen.2026.5599 (PMC13058769; doi:10.1001/jamanetworkopen.2026.5599)
Supplement: Supplement 2. — Data Sharing Statement [file jamanetwopen-e265599-s002.pdf]

Data Sharing Statement

Data
Data available: No

Additional Information
Explanation for why data not available: Data sharing not permitted by IRB.
